# Target–Distractor Competition Modulates Saccade Trajectories in Space and Object Space

**DOI:** 10.1523/ENEURO.0450-22.2023

**Published:** 2023-06-09

**Authors:** Caroline Giuricich, Robert J. Green, Heather Jordan, Mazyar Fallah

**Affiliations:** 1 School of Kinesiology and Health Science, York University, Toronto, Ontario M3J 1P3, Canada; 2 Centre for Vision Research, York University, Toronto, Ontario M3J 1P3, Canada; 3 Vision: Science to Applications (VISTA), York University, Toronto, Ontario M3J 1P3, Canada; 4 Department of Human Health and Nutritional Sciences, College of Biological Science, University of Guelph, Guelph, Ontario N1G 2W1, Canada

**Keywords:** attention, competiton, object representation, saccade trajectory, suppressive surrounds, target selection

## Abstract

Saccade planning and execution can be affected by a multitude of factors present in a target selection task. Recent studies have shown that the similarity between a target and nearby distractors affects the curvature of saccade trajectories, because of target–distractor competition. To further understand the nature of this competition, we varied the distance between and the similarity of complex target and distractor objects in a delayed match-to-sample task to examine their effects on human saccade trajectories and better understand the underlying neural circuitry. For trials with short saccadic reaction times (SRTs) when target–distractor competition is still active, the distractor is attractive and saccade trajectories are deviated toward the distractor. We found a robust effect of distance consistent with saccade vector averaging, whereas the effect of similarity suggested the existence of an object-based suppressive surround. At longer SRTs, there was sufficient time for competition between the objects to complete and the distractor to be repulsive, which resulted in saccade trajectory deviations away from the distractor exhibiting the effects of a spatial suppressive surround. In terms of similarity, as the target–distractor similarity decreased, the initial saccade angle shifted toward the target, reflecting stronger distractor inhibition. There were no interactions between distance and similarity at any point in the time course of target–distractor competition. Together, saccade trajectories reflect target–distractor competition that is affected independently by both spatial and object space suppressive surrounds. The differences in saccade trajectories at short and long SRTs distinguish between active and completed decision-making processes.

## Visual Abstract

**Figure:**
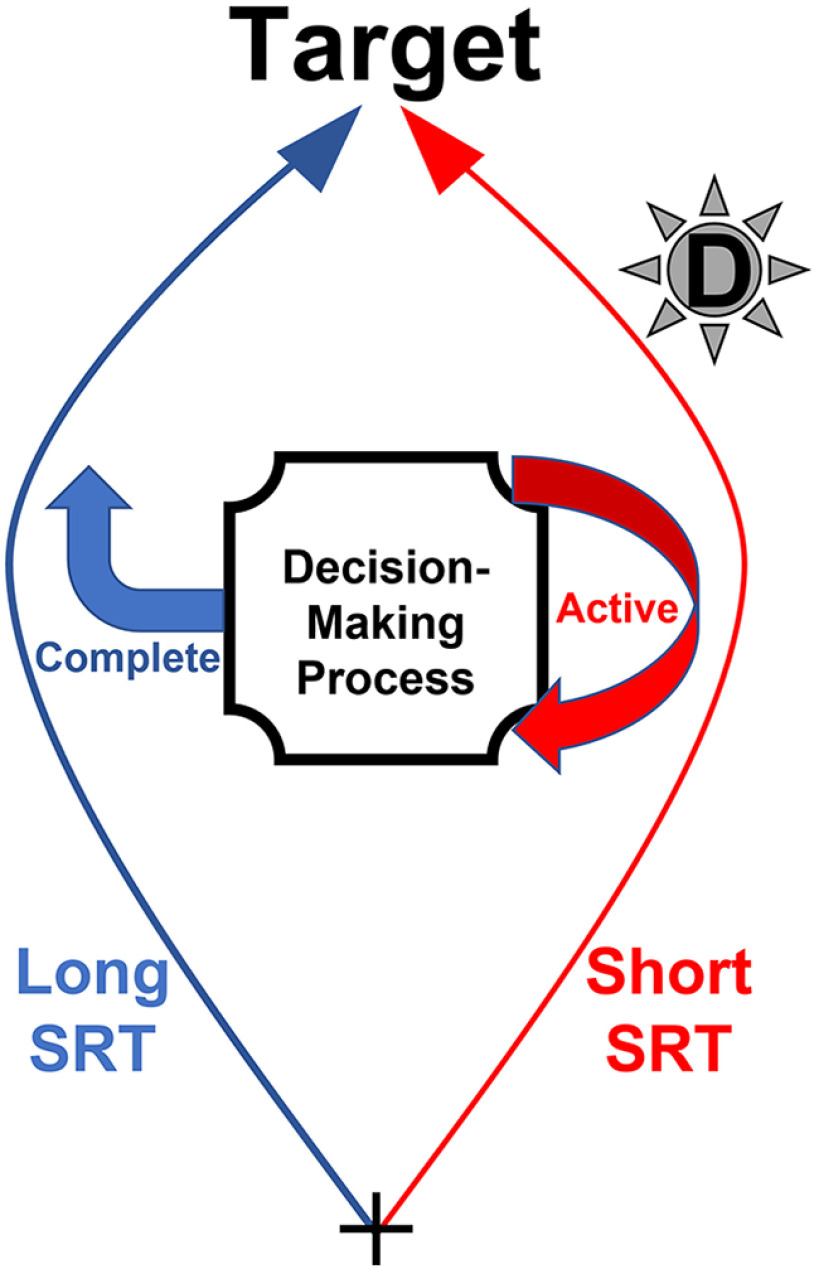

## Significance Statement

This is the first study to determine that the distance and similarity between visual objects independently affect saccade trajectories driven by the target–distractor competition process. Thus, spatiotemporal and object identity factors separately feed into saccade planning and execution, resulting in modulations of saccade trajectory metrics, which are based on spatial and object space suppressive surround mechanisms. Furthermore, this modulation of trajectory metrics distinguishes between active and complete decision-making processes. The findings are important for understanding the dynamic networks guiding target selection and are relevant for further development of decision-making models, as well as eye-tracking applications in health and disease.

## Introduction

Two potential saccade goals in the environment compete for attention with the saccade made to the target curving toward the interesting distractor (Findlay and Harris, 1984; Van Gisbergen et al., 1987; Minken et al., 1993; McPeek and Keller, 2001; McPeek et al., 2003; Port and Wurtz, 2003). Curvature toward the distractor results from unresolved target–distractor competition related to the neural processing of both objects in the superior colliculus and frontal eye fields (McPeek et al., 2003; McPeek, 2006) because of distractor neural activity increasing above baseline (McPeek and Keller, 2001; McPeek et al., 2003; Port and Wurtz, 2003). With enough time between stimulus onset and saccade initiation, target–distractor competition fully resolves, suppressing the distractor neural activity below baseline causing saccades to curve away instead (Doyle and Walker, 2001, 2002; McSorley et al., 2004; White et al., 2012). Saccadic reaction time (SRT) plays a large role during target selection; shorter SRTs result in curvature toward the distractor, and longer SRTs result in curvature away from the distractor (Theeuwes and Godijn, 2004; McSorley et al., 2006; Walker et al., 2006; Mulckhuyse et al., 2009; Hickey and van Zoest, 2012). Putative mechanisms of active target–distractor competition and, when completed, distractor inhibition result in attraction and repulsion of saccade trajectories.

The similarity between competing objects can affect the saccade curvature in a target selection task. This demonstrates how bottom-up feature processing and top-down task demands are integrated into a priority map of the visual field. Studies have investigated the effect of similarity on saccade trajectories by varying low-level features like color and orientation; there is more curvature toward a distractor that is color congruent than incongruent (Ludwig and Gilchrist, 2003; Mulckhuyse et al., 2009). Similarity effects also occur for complex objects, as Kehoe et al. (2018b) found that the more similar the distractor, the less the saccade curvature. They suggested that the oculomotor system reweights competing saccade goals, putting the strongest weight on the most behaviorally relevant object, with distractors receiving weights based on similarity to that target. When targets and distractors can be distinguished by features or complex shapes, these weighted vectors are integrated to produce the final saccade trajectory.

Studies investigating the effect of a distractor on saccade trajectories often keep the distractor at a set distance to the target without varying similarity, treating the distractor as a placeholder for competing motor plans (Sheliga et al., 1995). To understand the interplay of distance and similarity on saccade trajectories, we simultaneously varied the angular distance (AD) in an egocentric reference frame and the similarity between a target and distractor in a delayed match-to-sample task. We predicted that the effects of distance on saccade trajectories would be more complex than a linear relationship because of putative mechanisms producing attentional suppressive surrounds, shown to follow a difference of Gaussian (DoG) pattern as predicted by the Selective Tuning model of visual attention (Tsotsos, 1990; Tsotsos et al., 1995; Cutzu and Tsotsos, 2003; Hopf et al., 2006; Boehler et al., 2009). A close distractor falls into the attentional spotlight, centered on the target. As the distractor is placed further away from the target, it is suppressed according to a gradient with the most suppression at a medium distance, caused by pruning connections irrelevant to the stimulus of interest (Cutzu and Tsotsos, 2003; Yoo et al., 2018). This suppression is reduced as distance increases until it disappears outside the range of the suppressive surround.

Attentional suppressive surrounds have also been found in feature space for basic features such as color, orientation, and direction of motion (Tsotsos et al., 2005; Tombu and Tsotsos, 2008; Störmer and Alvarez, 2014; Yoo et al., 2018). For example, as the orientation of a distractor shifts away from that of the target, attention follows a feature-based suppressive surround (Tombu and Tsotsos, 2008). We investigated whether object-based suppressive surrounds exist when attending to higher-order complex objects.

In the present study, we investigated how spatial distance, similarity, and the interplay between them affect saccade programming through examining spatial and object-based suppressive surrounds. We hypothesized that with enough time for target–distractor competition to be resolved, the distractor would be suppressed according to a spatial suppressive surround with a DoG-shaped effect on saccade trajectory deviations. When varying the target–distractor similarity, we expected that if an object-based suppressive surround exists, we would find a DoG modulation of saccade trajectory deviations, but that this would be limited by distance since similarity computations depend on local competition within a given visual area.

## Materials and Methods

### Participants

Twenty-six participants (age range, 18–43 years; 6 males) took part in this experiment, all with normal or corrected-to-normal vision. Participants were naive to the purpose of the experiment and received partial course credit for participating, when applicable. They provided written informed consent before beginning the experiment. Human subjects were recruited at York University, and the York University Human Participants Review Committee approved this study.

### Stimuli

**Figure 1.**
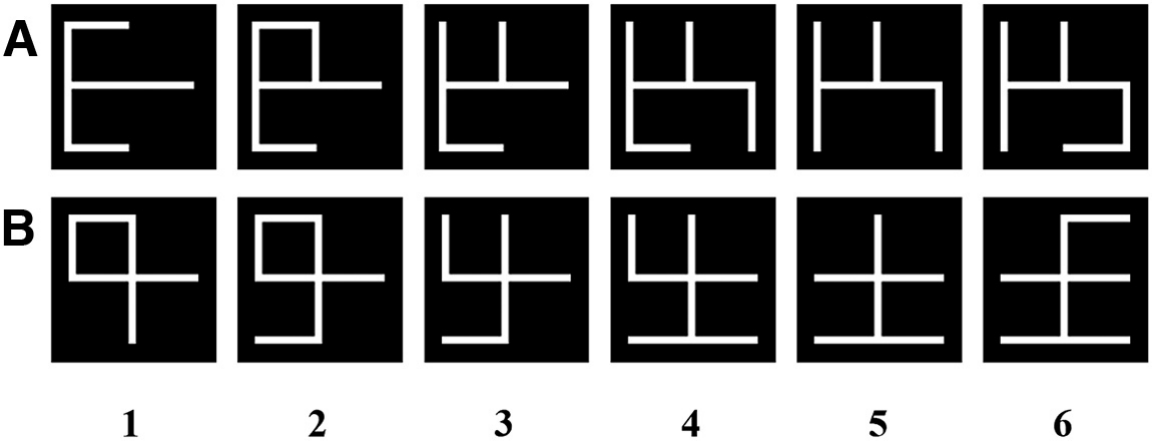
***A***, ***B***, Stimulus sets. The stimuli were numbered from 1 to 6 (left to right). The difference between stimulus numbers gives rise to the number of line differences (objective similarity levels).

The stimuli used were developed in-house using MATLAB (MathWorks) and were based on an earlier study (Kehoe et al., 2018b). They consisted of a combination of six or seven vertical and horizontal line segments (1° × 0.08°) positioned orthogonal to each other. This number of line segments was used to ensure holistic representations of the objects rather than depending on human visual working memory capacity (Luck and Vogel, 1997; Kehoe et al., 2018b). These stimuli were created to be novel to participants, and thus do not include letters or numbers in the English language. Line segments were white on a black background (white: CIExy (International Commission on Illumination xy color space), [0.29, 0.30]; luminance, 126.02 cd/m^2^; black: CIExy, [0.27, 0.26]; luminance 0.20 cd/m^2^) and were put together to fit within a 2° × 2° box. Two sets of stimuli were used and shuffled throughout the experiment (Fig. 1).

### Apparatus

Experimental control was maintained with Presentation software (Neurobehavioral Systems). Participants ran the experiment in a dark room on a 21 inch CRT monitor (60 Hz; 1024 × 768), 57 cm away from a head and chin rest. Eye position was recorded from the left eye using an infrared eyetracker (500 Hz; EyeLink II, SR Research). Eye position was calibrated at the start of the experiment and during the experiment, as necessary. Participants responded via a serial response box (6 participants; Cedrus) or a mouse (20 participants; Dell).

### Procedure

**Figure 2.**
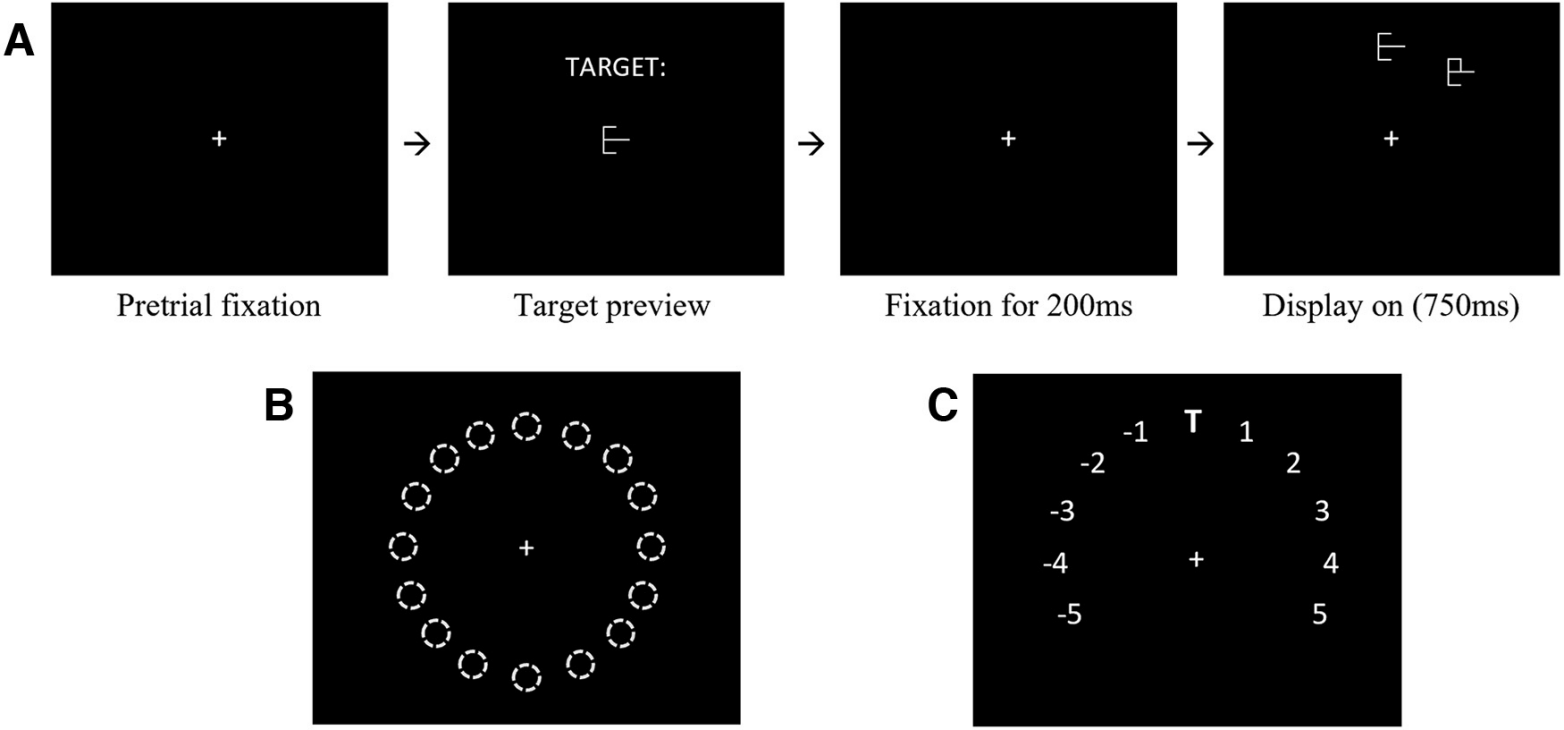
Task paradigm and display layout. ***A***, Task paradigm. Participants fixated on the central cross and pressed a button to move to the target preview. Once ready, they pressed the button again to start the search. Participants stabilized fixation for 200 ms, then the display came on. The target and distractor remained visible until the participant fixated on an object or until 750 ms had passed. ***B***, Sixteen possible object locations, 22.5° apart, at an eccentricity of 8 dva. The target was randomly placed at one location, and the distractor was placed relative to the target on the clockwise or counterclockwise side. ***C***, AD locations relative to the target. With the target (T) at the top location as an example, the distractor could be placed from 1 to 5 positions clockwise, or from 1 to 5 positions counterclockwise.

We used a delayed match-to-sample task where participants needed to search for a previously shown target object from a distractor object after a short delay from target preview. Participants started each trial when shown a small, white fixation cross (0.4° × 0.4°) at the center of the screen by pressing a button (serial response box, center button; mouse, left button). The target object was then previewed on the screen (Fig. 2*A*) until the participant pressed the button to indicate they were ready to begin the trial. Upon button press, the preview was replaced with a central fixation cross. After fixating the cross for 200 ms, the target and distractor objects appeared simultaneously at isoeccentric points around a circle of radius 8° of visual angle (dva). Participants were instructed to use their peripheral vision to identify the target while maintaining central fixation and respond by moving their gaze to the target. The trial ended when an eye movement was made within a 1.9 dva square box around the target or distractor, or after 750 ms if no movement had been made. These time-out trials were reshuffled back into the remaining trial array. An error tone and message were used to indicate incorrect or time-out trials.

The target and distractor were placed at any of 16 equally spaced positions around the circle (Fig. 2*B*). The target was randomly placed at one location, and the distractor was placed relative to the target on either the clockwise or counterclockwise side. Similarity and angular distance between the target and distractor were varied and pseudorandomized for each trial. Lone-target trials without a distractor were considered baseline trials and comprised 20% of the total. This resulted in 50 trials per block, with 10 blocks for a total of 500 trials. Before block 1, participants were given 10 practice trials, which were not included in the analysis. The experiment took ∼45 min to complete. All participants included in the analysis completed the full 500 trials.

### Similarity and angular distance

Objective similarity (OS) was determined by the number of line segment differences between two given stimuli, defined by adding or removing individual line segments (Fig. 1). Target similarity differences were confined to the OS range 1–4. AD was calculated as the absolute difference between target and distractor locations in polar angle (Fig. 2*B*,*C*). An AD range of ±1–5 locations (spread out by 22.5°) was used in this experiment (Fig. 2*C*).

### Analysis

#### Task performance

The behavioral accuracy score is the proportion of trials where the participants correctly made a saccade to the target object. A binomial test was performed on the behavioral accuracy scores for each participant to test that they were more accurate than chance (50%). No participants were excluded from analysis since all scores were significantly better than chance.

#### Saccade detection

In-house written MATLAB scripts were used to filter saccades for each trial. We defined saccades as having a peak velocity >50°/s and, for at least 8 ms, a velocity that was >20°/s. Only correct saccades in which the participants made a saccade to the target were included for analysis. The first saccade of each trial was analyzed, and trials in which corrective saccades were needed to reach the target or blinks occurred were excluded from analysis. Saccades with an amplitude <1° (1.26%) or with an SRT <100 ms (2.96%) were also excluded. In total, 83.82% of correct saccades were included for further analysis.

#### Saccade metric calculations

Five saccade metrics were calculated for each saccade included in the analysis, as follows: initial angle, end point deviation, sum curvature, maximum (max) curvature, and angle at maximum curvature. For all saccade metric calculations, the starting position of the saccade was shifted to the origin and rotated so that the target position was on the positive *y*-axis at 8 dva. The initial angle was calculated as the angle between the line from the start of the saccade to the target (i.e., the *y*-axis) and the point 20% along the saccade trajectory. End point deviation was calculated as the angular difference between the last point of the saccade trajectory and the target (i.e., the *y*-axis). The curvature metrics were calculated with the saccade rotated so that both the start and end points lay on the positive *y*-axis. Sum curvature was calculated as the sum of all *x*-values from the points along the saccade trajectory. Max curvature was the *x*-coordinate value of the point with the maximum absolute *x*-value along the saccade trajectory. The angle at max curvature was the angular difference between a straight saccade (i.e., the *y*-axis) and the max curvature coordinate. These metrics were baseline subtracted using the average lone-target (distractor-absent) trial metrics at the corresponding target location of that trial. Saccade metrics calculated from trials with negative ADs (counterclockwise placement of distractor relative to target) were reflected and collapsed with the positive AD metrics of clockwise distractors. This kept the distractor on the positive *x* side of the *y*-axis and resulted in five total ADs for analysis. All positive metrics signify deviations toward the distractor, and negative metrics signify deviations away from the distractor. A value of 0 for a saccade metric represents a trajectory where the distractor had no effect as it matched the lone-target/distractor-absent condition.

#### Saccade target onset asynchrony

Saccade target onset asynchrony (STOA) was calculated as the time between target onset and saccade initiation, where saccade initiation was taken to be at 0 ms. STOA values were averaged over a sliding window of 10 ms from −500 to −100 ms. Time-averaged metric data were plotted over STOA where each point was an average of the metric data from trials with SRTs that fell within the sliding 10 ms windows used to calculate STOA. Trials with SRTs >500 ms (2.5% of total trials) were treated as outliers that were not representative of the rest of the data and were not included in further analysis.

#### Average saccade trajectories

Average saccade trajectories were calculated and plotted using prior published techniques (Moehler and Fiehler, 2017). The *x*-/*y*-coordinates of saccades were separated by OS and/or AD and averaged together by first shifting the trajectory so that the first point was at the origin, and then taking points within 10% sections of the trajectory. Those points were averaged together to create an average saccade to visualize the calculated metrics. We baseline corrected the *x*-coordinates of the trajectories by subtracting the values from the average lone-target trial trajectory of the same trial location. Shaded regions around each average trajectory represent 1 SEM.

#### Metric plots and curve fits

Averages of the five metrics were plotted across the four OS levels and five ADs. The SEM was calculated and plotted as error bars. We fit each plot with linear, quadratic, sigmoid, Gaussian, DoG, and exponential curves. We assessed the goodness of fit using the coefficient of determination (*R*^2^), Akaike information criterion (AIC), and the *p*-value associated with the *F* test of the regression analysis. Our experimental paradigm did not support the use of a DoG curve fit since we did not have the full range of data to represent the excitatory peak of a DoG curve that would occur between our +AD and –AD conditions. Thus, a single Gaussian fit was used to model the suppressive effect over collapsed ADs. Data were also split by OS across ADs to determine the effect of OS at each AD. For each metric, a five AD × four OS univariate ANOVA was performed in SPSS (version 27; IBM) for short and long SRTs separately. Scheffé’s test *post hoc* analysis was used to investigate significant effects across conditions.

## Results

### Behavioral accuracy

Each participant’s behavioral accuracy was significantly above chance, with a mean accuracy across participants of 74%. Only correct trials where the participants made a saccade to the target were included in analysis.

### Individual saccade traces and saccade metric measurements

**Figure 3.**
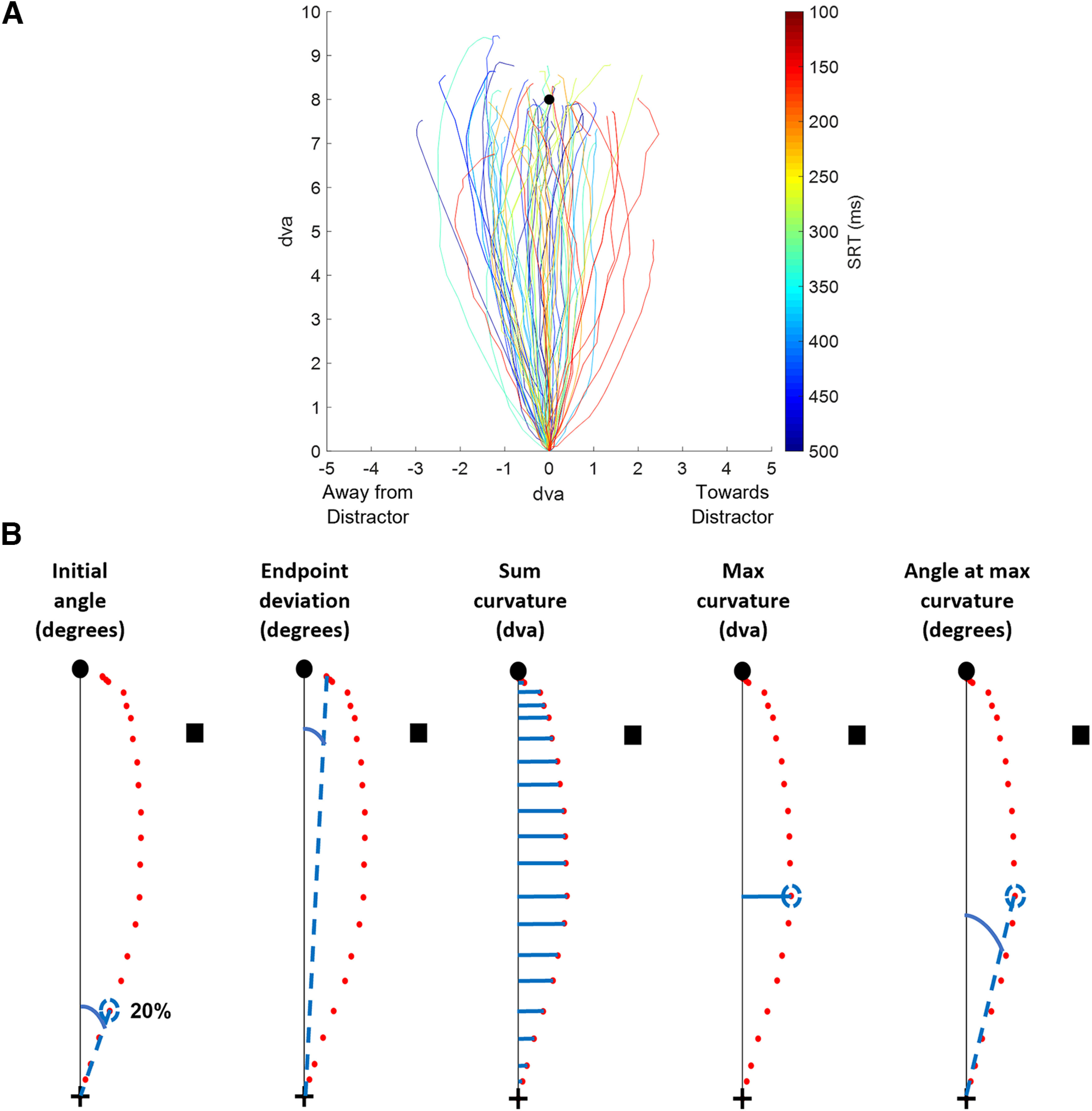
Individual saccade traces and saccade metric measurements. ***A***, Eighty random individual saccades from the angular distance 3 condition collapsed across objective similarity colored by SRT, as shown by the colorbar. The darkest blue was assigned to trials with SRTs closest to 500 ms, and the darkest red was assigned to trials with SRTs closest to 100 ms. The black circle represents the target position at 8 dva. ***B***, Red dots represent eye position coordinates from a saccade trajectory recorded at 2 ms intervals. The black circle and square represent the target and distractor, respectively, and the black cross represents central fixation. Solid blue lines represent the metric itself, whereas dashed blue lines represent the depiction of the calculation. The trajectory is rotated for the curvature metrics so that the final point of the saccade lies on the vertical line connecting the fixation cross to the target position.

Saccade trajectories were delineated by eye position coordinates recorded every 2 ms (500 Hz) that started at the central fixation cross and ended near the target (see Materials and Methods). To visualize saccade trajectories produced in our task, we plotted a random selection of 80 individual saccades taken from the condition AD = 3 (including all OS levels) in Figure 3*A* using their *x*-coordinates and *y*-coordinates (in dva). The saccades were adjusted as described in Materials and Methods for better visualization of the saccade metrics, as follows: they were shifted so the starting point was at the origin, and rotated so that all target locations were positioned at the top and the *x*-coordinates of trials with negative ADs were rectified and collapsed with those of positive ADs. The saccades were colored by SRT from short to long using a heatmap color range from dark red (100 ms) to dark blue (500 ms). This plot showed that, given a random selection of trials, the longer SRTs generally showed trajectory shifts away from the distractor (negative deviations from a vertical line between the starting point and the target) and the shorter SRTs generally showed trajectory shifts toward the distractor (positive deviations from a vertical line).

We measured deviations in the saccade trajectory made to the target using five saccade metrics: initial angle, end point deviation, sum curvature, max curvature, and angle at max curvature. Figure 3*B* depicts the measurements of these metrics using an example saccade trajectory that curves toward the distractor. The initial angle and end point deviation demonstrate the direction of the saccade vector at the start and end of the trajectory. The sum, max, and angle at max curvatures demonstrate the extent of the deviation of the saccade toward the distractor (positive deviations) or away from the distractor (negative deviations). The five metrics derived from the saccades were then used in later analyses of angular distance and objective similarity.

### Saccade target onset asynchrony and classification of saccadic reaction times

**Figure 4.**
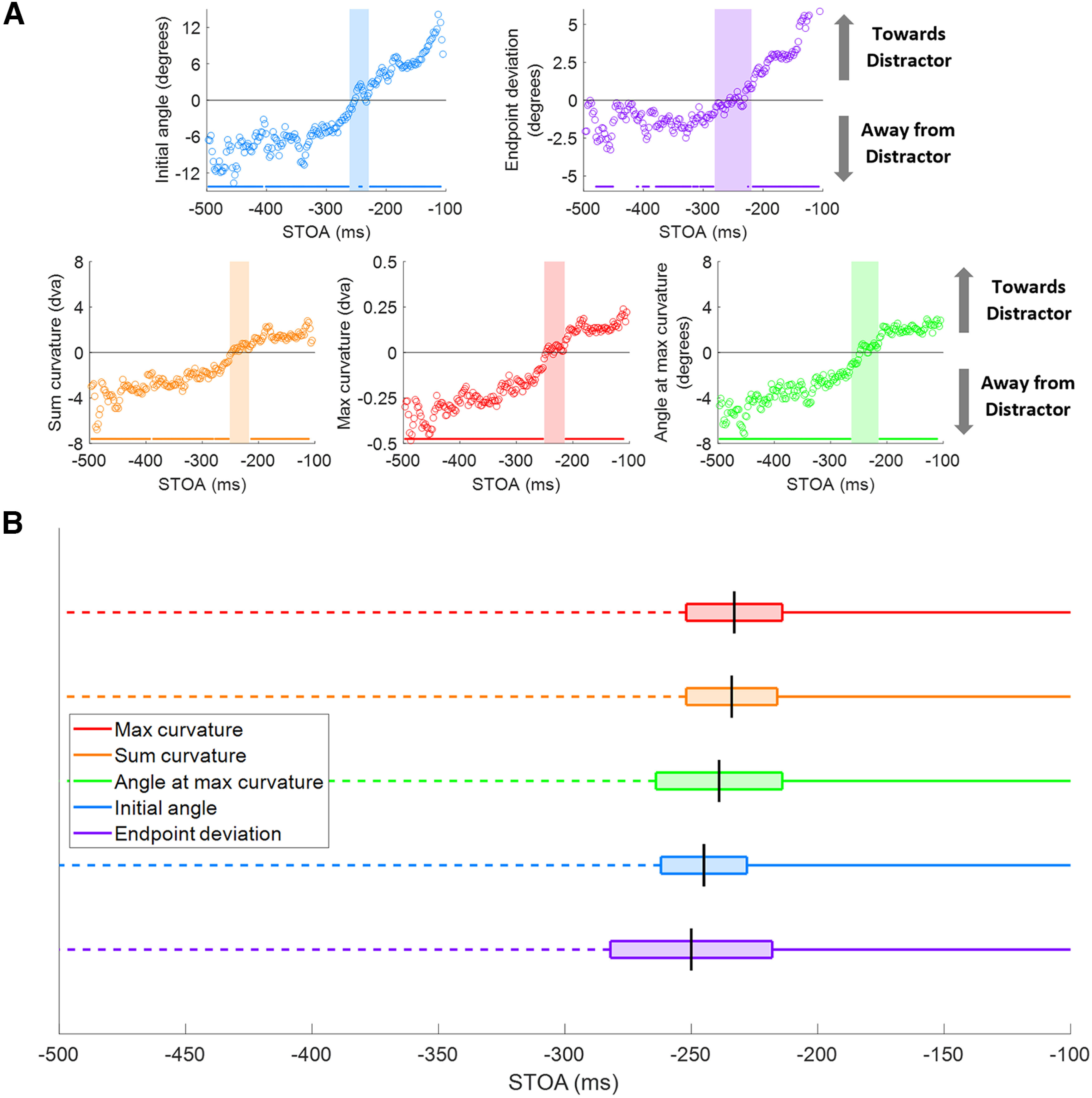
Metrics over time and transition period ranges. ***A***, Metrics plotted over STOA. Points were calculated by averaging data over a 10 ms window. Colored dots above the *x*-axis represent significant deviations from zero, as calculated through *t* tests performed on each point. Gray arrows pointing up represent deviations toward the distractor, and gray arrows pointing down represent deviations away from the distractor. Shaded colored regions represent the transition period for each metric that was removed from analysis because of those points not being significantly deviated from zero. ***B***, Transition period ranges by metric over SRT, ordered by midpoint from earliest to latest. The transition period is represented by the box and the midpoint is shown with a black vertical line. Each solid-colored line represents the short SRT period where that metric significantly deviated toward the distractor. Each dashed-colored line represents the long SRT period where that metric significantly deviated away from the distractor.

As prior studies have shown that distractor competition in oculomotor planning shifts from an early attraction to the distractor to later repulsion away from the distractor (Theeuwes and Godijn, 2004; McSorley et al., 2006; Walker et al., 2006; Mulckhuyse et al., 2009; Hickey and van Zoest, 2012), we first wanted to determine the timing of these effects empirically from the data in our paradigm. All five metrics were plotted against STOA values (Fig. 4*A*) to visualize the switch from trajectory deviations toward the distractor (positive deviations) to away from the distractor (negative deviations). This plot shows the time-averaged metrics with *t* test comparisons of each point to zero, where significant deviations are represented by dots above the *x*-axis. For each metric, the middle range of points that were not significantly different from zero was defined as the “transition period” of that metric where oculomotor planning did not produce clear positive or negative deviations. It should be noted that as STOA approaches –500 ms, the number of trials in each 10 ms window approaches zero, leading to more variability in the average metric data. We used these results to classify trials by their SRTs into short, long, and transition periods (Fig. 4*B*) where significant positive deviations toward the distractor occurred for short SRTs, and significant negative deviations away from the distractor occurred for long SRTs. The SRTs in the transition period did not produce saccades that were significantly different from zero for each metric (Table 1, ranges). To test whether the transition period consisted of proportional mixtures of significant positive and negative deviations resulting in a broad bimodal distribution that overlapped zero or a unimodal distribution of saccades that were not significantly deviated by distractors, the metric data from trials with SRTs within the transition periods were plotted as histograms and fit with single and dual Gaussians. Each metric histogram fit well with a single Gaussian distribution compared with a dual Gaussian, all with *R*^2^ > 0.96 and *p* < 0.001. Therefore, the transition periods reflect the oculomotor system switching from an excitatory to an inhibitory drive for distractor competition. The saccade metrics were ordered by the midpoint of each of their transition periods from earliest to latest (Fig. 4*B*). The curvature metrics reached the transition period earlier than the angle metrics. All subsequent analyses were performed separately for short and long SRTs outside of the transition periods. It is important to note that trials with short SRTs made up 55% of total trials included in the analysis, whereas trials with long SRTs made up 30%. When computing the average saccade trajectory, we excluded only trials that fell within the transition zone for all metrics (Table 1).

**Table 1:** Short SRT ranges, transition period, and long SRT ranges (ms) for each metric and the average saccade analysis

	Short SRT range	Transition period range	Long SRT range
Initial angle	100–228	230–260	262–500
End point deviation	100–218	220–280	282–500
Sum curvature	100–216	218–250	252–500
Max curvature	100–214	216–250	252-500
Angle at max curvature	100-214	216–262	264–500
Average saccades	100-228	230–250	252-500

**Figure 5.**
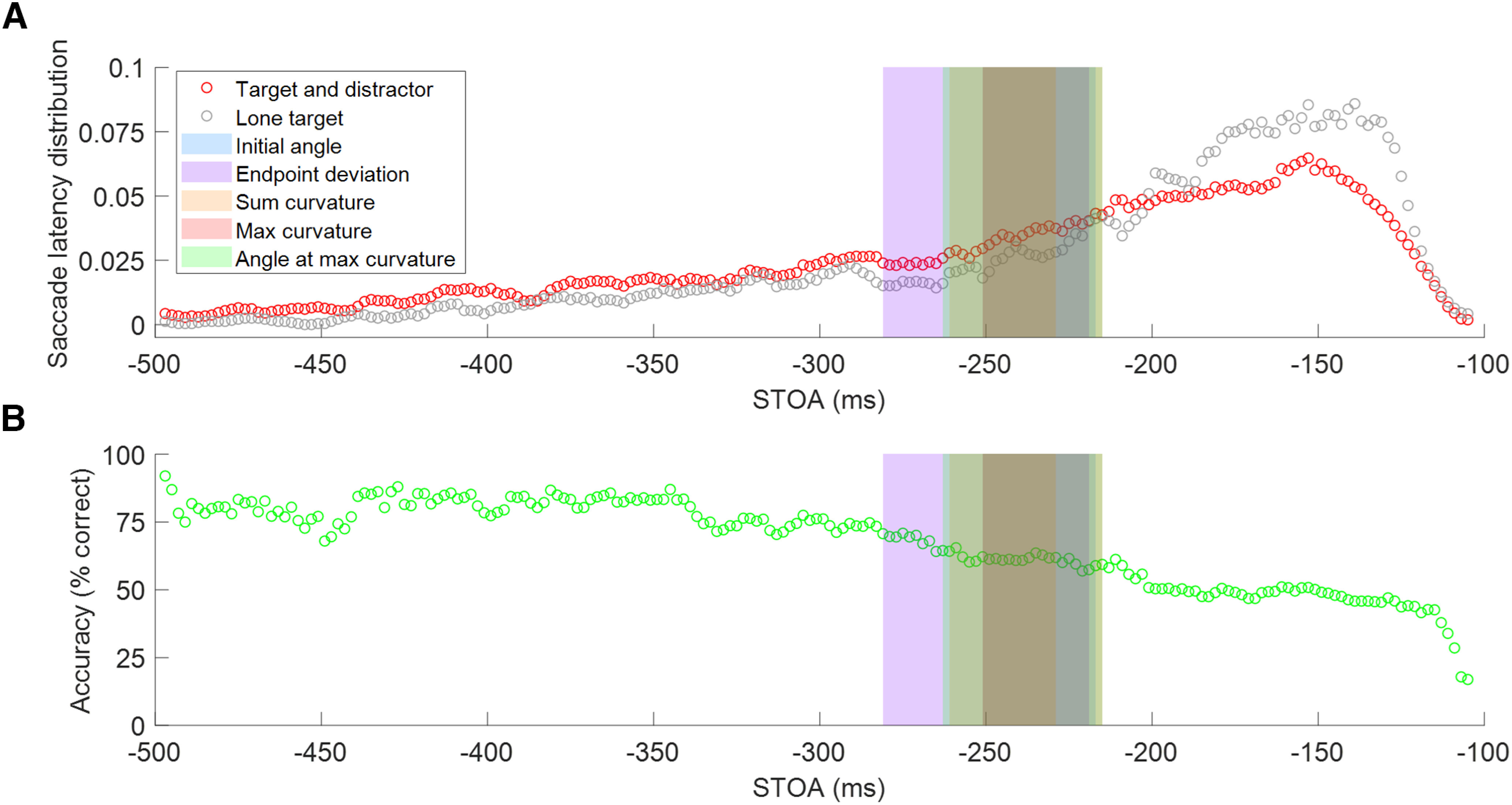
Saccade latency distribution and accuracy. Shaded colored regions cover the transition period for each metric that was removed from analysis. ***A***, Saccade latency distribution plotted in red over STOA as a proportion of total trials. Saccade latency distribution plotted in gray for lone-target (no distractor) trials. ***B***, Accuracy plotted over STOA as a percentage of total trials.

To better understand the time course of the visual search task, we plotted the saccade latency distribution (the number of trials within a given 10 ms sliding window in which a saccade was initiated as a proportion of total correct trials) over STOA (Fig. 5*A*, red). This showed that the peak frequency of saccade initiations occurred at ∼150 ms, as would be expected with visually evoked saccades. After this peak, there was a general decrease in saccade initiation frequency as STOA increased. The saccade latency distribution for lone-target trials (no distractor, in gray) similarly showed a peak frequency of ∼150 ms followed by a general decrease, slightly steeper than the distribution for trials with a distractor. As mentioned for Figure 4*A*, as STOA gets closer to –500 ms in the long SRT period, the number of trials in a given sliding window decreases, causing increases in variability in the metrics. Accuracy over STOA (plotted as the percentage of trials that participants made a saccade to the target as a proportion of all total trials) showed that the longer the participants took to process the display, the less likely they were to incorrectly make a saccade to the distractor object, as reflected in the STOA (Fig. 5*B*). There was also more variance in accuracy at long SRTs. These results show that as SRT increases from short to long, saccade trajectories made to the target shift from attraction to repulsion of the distractor, with the highest frequency of trials occurring at short SRTs, but more accurate trials occurring at long SRTs. These results also provide a measure of how long active discrimination of the target and distractor occurs before the target is identified and the distractor is inhibited, which then repulses the saccade trajectory.

### Average saccades

**Figure 6.**
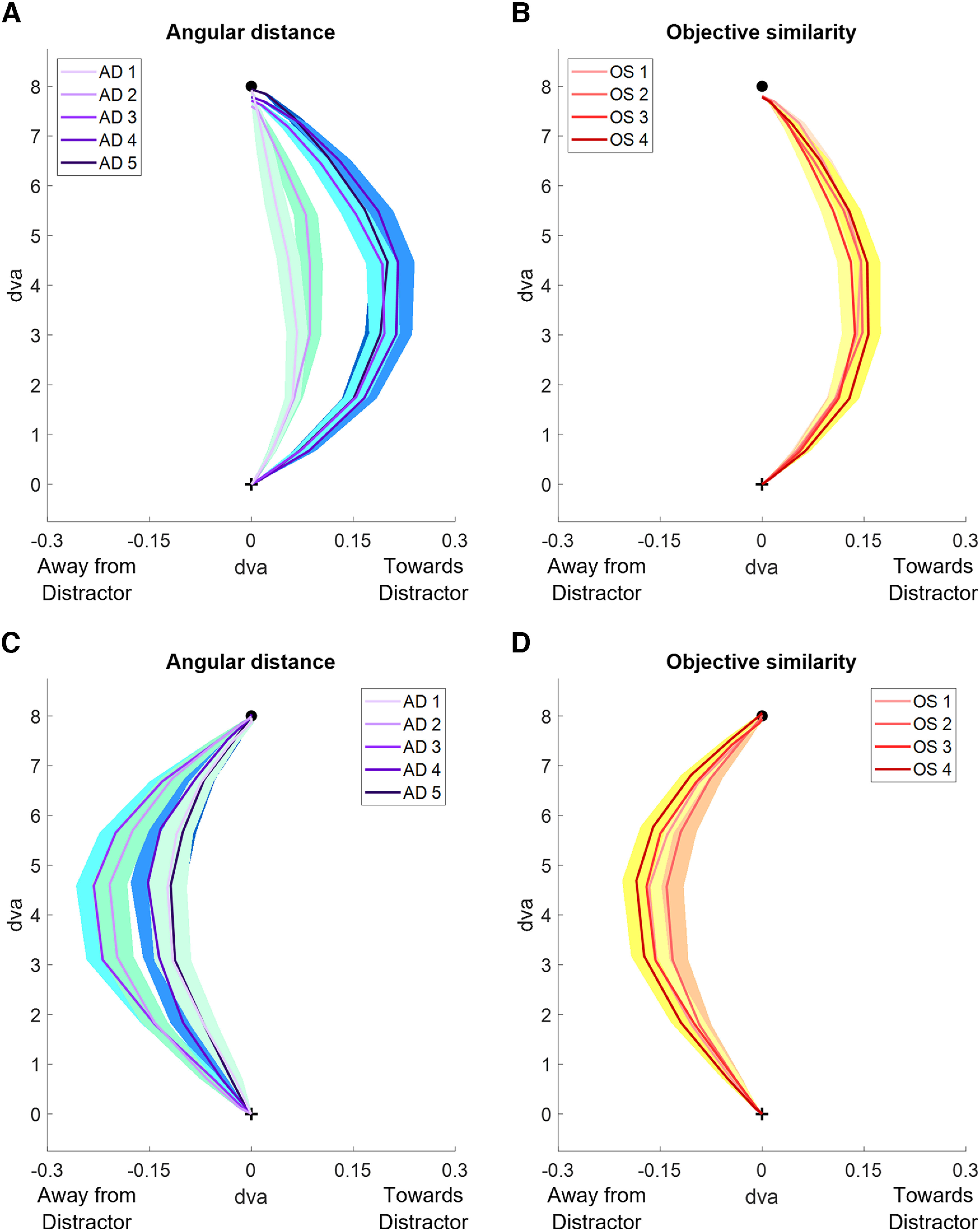
Average saccade plots for short and long SRTs split by AD and OS. Shaded regions represent the SEM for each average point along the trajectories. The black cross represents the fixation point. The black circle represents the target position at 8 dva. ***A***, Average saccades for each AD for short SRTs. ***B***, Average saccades for each OS for short SRTs. ***C***, Average saccades for each AD for long SRTs. ***D***, Average saccades for each OS for long SRTs.

Average saccade trajectories were plotted across AD and OS for short and long SRTs (Fig. 6) to depict the shape of saccades made to the target for each condition during both SRT periods. The average saccades showed saccades deviated positively (toward the distractor) for short SRTs versus negatively (away from the distractor) for long SRTs. When looking at the differences between OS levels and ADs, distance had a greater effect on saccade trajectory than did similarity for short SRTs. We investigated whether this could be because of the angular distance between the target and distractor limiting the trajectory deviation toward the distractor (but not away from the distractor). Figure 6*A* qualitatively shows a clear distinction between the average trajectories for AD = 1, 2 compared with AD = 3, 4, 5 in the initial angle and amount of curvature toward the distractor. This suggests that it is not simply a result of the distance limiting the trajectory deviation, as there is a plateau across ADs 3–5. On the other hand, Figure 6*B* shows that the average trajectories for each OS were clustered together. For long SRTs, the angular distance variations (Fig. 6*C*) showed evidence of a spatial suppressive surround; the average saccade trajectories away from the distractor were more deviated for AD = 2, 3 than for AD = 1, 4, 5. In comparison, the objective similarity differences (Fig. 6*D*) showed that the average saccade trajectories were more spread out between each OS compared with those at short SRTs (Fig. 6*B*). The average saccade trajectories displayed that short SRTs produced deviations toward the distractor while long SRTs produced deviations away from the distractor, and that angular distance modulated saccade trajectories to a greater extent than similarity.

### Angular distance effects

**Figure 7.**
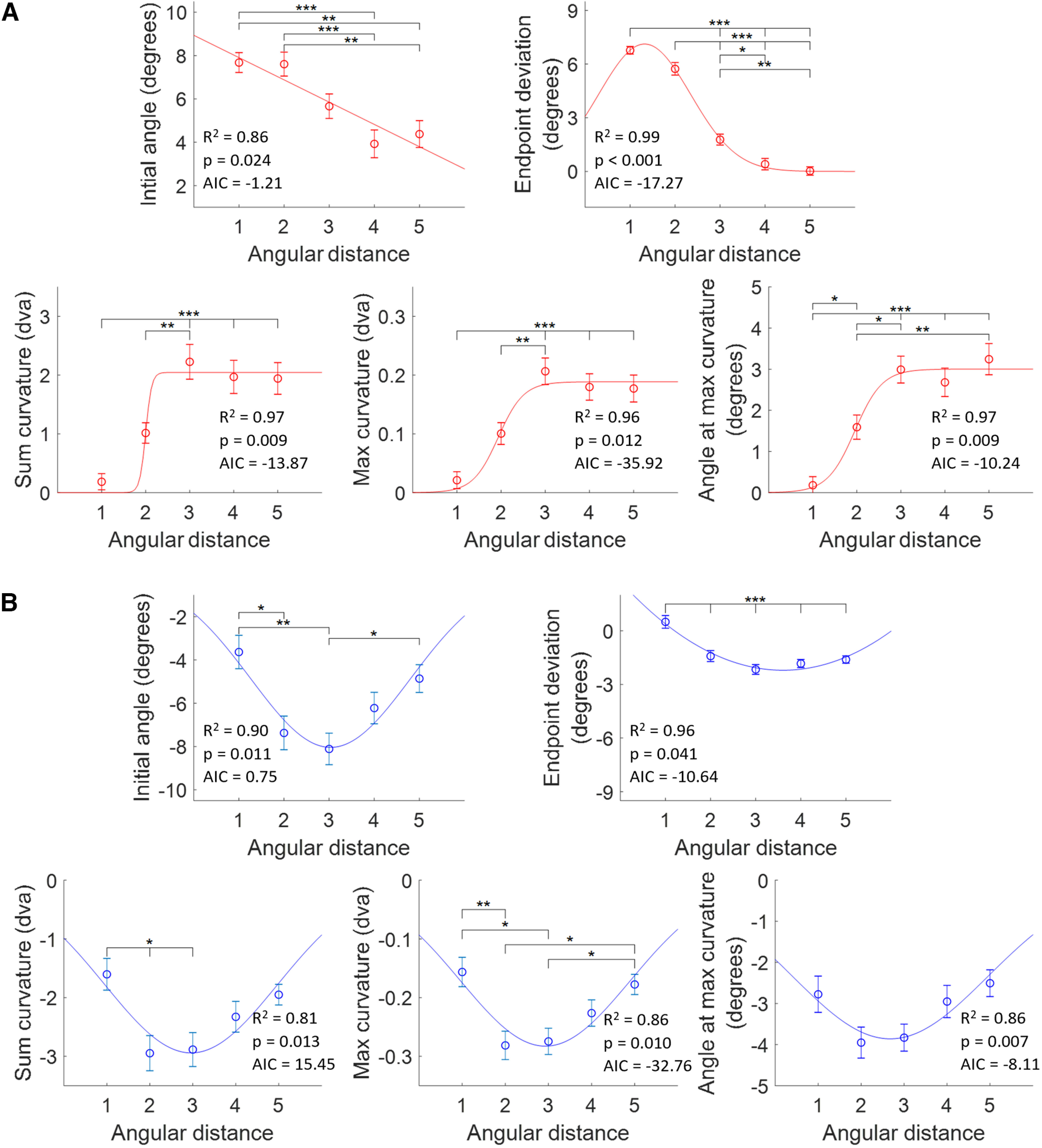
Saccade metric averages over AD with curve fits for short and long SRTs. Metric values were averaged across all objective similarity levels. Curve fits were chosen according to goodness-of-fit metrics (*R*^2^, AIC, *p*). Plots include the respective *R*^2^, *p*-values, and AIC. Error bars are SEM. Brackets indicate significant differences (*p < 0.05, **p < 0.01, ***p < 0.001) from Scheffé’s test *post hoc* analysis. ***A***, Metrics over AD for short SRTs. Curve fits from left to right: top, linear, sigmoid; bottom, sigmoid, sigmoid, Gaussian. ***B***, Metrics over AD for long SRTs. Curve fits were all Gaussian except for end point deviation, which was fit with a quadratic.

To determine the effects of distance and similarity on saccade trajectories, we subjected each metric to a two-way ANOVA (AD × OS) separately for short and long SRTs. The results are shown in Table 2. As there was a main effect of AD on all metrics at short SRTs, we next performed Scheffé’s *post hoc* tests on each metric for short SRTs (Table 3) to see at which ADs the metrics significantly differed, which is depicted in Figure 7 (where the individual metrics are averaged at each AD across all OS levels). For long SRTs, there were main effects of AD on all metrics, as well (Table 2). Scheffé’s *post hoc* tests were performed for AD versus metrics for long SRTs (Table 3) and, again, is depicted in Figure 7.

**Table 2:** AD and OS Main effect ANOVA *p*-values for each metric for short and long SRTs

	Short SRTs		Long SRTs	
Saccade Metric	AD	OS	AD	OS
Initial angle	< 0.001***^†^	0.606	< 0.001***^†^	0.052
End point deviation	< 0.001***^†^	0.311	< 0.001***^†^	0.020*
Sum curvature	< 0.001***^†^	0.746	0.001**^†^	0.470
Max curvature	< 0.001***^†^	0.831	< 0.001***^†^	0.736
Angle at max curvature	< 0.001***^†^	0.658	0.020*	0.267

*p < 0.05, **p < 0.01, ***p < 0.001.

^†^Scheffé’s test *post hoc* analysis showed significant differences between conditions.

**Table 3:** Significant Scheffé’s test *post hoc p*-values between ADs for each metric for short and long SRTs

SRT period	Metric	AD		*p*-value
Short	Initial angle	1	4	<0.001
			5	0.002
	End point deviation	1	3	<0.001
			4	<0.001
			5	<0.001
		2	3	<0.001
			4	<0.001
			5	<0.001
		3	4	0.030
			5	0.002
	Sum curvature	1	3	<0.001
			4	<0.001
			5	<0.001
		2	3	0.008
	Max curvature	1	3	<0.001
			4	<0.001
			5	<0.001
		2	3	0.007
	Angle at max curvature	1	2	0.019
			3	<0.001
			4	<0.001
			5	<0.001
		2	3	0.032
			5	0.008
Long	Initial angle	1	2	0.023
			3	0.002
		3	5	0.025
	End point deviation	1	2	<0.001
			3	<0.001
			4	<0.001
			5	<0.001
	Sum curvature	1	2	0.023
			3	0.024
	Max curvature	1	2	0.009
			3	0.011
		2	5	0.025
		3	5	0.029

*p*-Values are significant at *p* < 0.05.

We fit each metric plot with linear, quadratic, sigmoid, Gaussian, DoG, and exponential curves to better understand the underlying neural mechanisms involved and the computations used to reflect distance in the target–distractor competition that produced the saccade motor plan. We used goodness-of-fit metrics (*R*^2^, AIC, *p*-values) to determine the function with the best fit for each metric (Table 4). All best fits had significant *p*-values (*F* test from the regression analysis). For long SRTs, as Gaussian and quadratic curves fit all metrics similarly, excluding end point deviation, we relied on Gaussian curves as they are often found for modeling neural mechanisms.

**Table 4:** *R*^2^, AIC, *p*-values for each curve fitted to metric plots over AD and OS for short and long SRTs

Condition	SRT period	Curve fit	Initial angle	End point deviation	Sum curvature	Max curvature	Angle at max curvature
AD	Short	Linear	0.86, −1.21, 0.024^†^	0.91, 1.96, 0.011	0.69, −4.55, 0.082	0.67, −28.38, 0.092	0.82, −3.27, 0.35
		Quadratic	0.87, 0.46, 0.134	0.94, −2.01, 0.058	0.94, −10.57, 0.063	0.94, −34.81, 0.062	0.95, −7.43, 0.053
		Sigmoid	0.87, 1.57, 0.013	0.99, −15.42, <0.001	0.97, −13.87, 0.009^†^	0.96, −35.92, 0.012^†^	0.97, −10.24, 0.009^†^
		Gaussian	0.87, 1.62, 0.013	0.99, −17.27, <0.001^†^	0.91, −7.90, 0.028	0.91, −31.90, 0.027	0.88, −2.36, 0.039
		DoG	0.90, 1.81, 0.099	0.95, 1.78, 0.033	0.94, −9.89, 0.019	0.94, −34.24, 0.017	0.96, −7.83, 0.013
		Exponential	0.86, −0.52, <0.001	0.88, 4.52, 0.013	0.55, −2.03, 0.029	0.54, −26.04, 0.027	0.69, 0.10, 0.018
	Long	Linear	0.01, 8.83, 0.856	0.49, −0.05, 0.741	0.00, −2.51, 0.974	0.00, −25.93, 0.955	0.14, −2.20, 0.534
		Quadratic	0.90, −0.52, 0.102	0.96, −10.64, 0.041^†^	0.81, −8.93, 0.186	0.86, −33.77, 0.140	0.82, −7.98, 0.181
		Sigmoid	0.55, 10.52, 0.046	0.91, −6.78, 0.006	0.50, −1.95, 0.004	0.44, −24.59, 0.004	0.14, 1.62, 0.041
		Gaussian	0.90, 0.75, 0.011^†^	0.77, −0.81, 0.115	0.81, 15.45, 0.013^†^	0.86, −32.76, 0.010^†^	0.86, −8.11, 0.007^†^
		DoG	0.00, 10.39, 0.002	0.91, −5.15, 0.048	0.40, 0.14, 0.250	0.00, −23.31, <0.001	0.00, 7.64, 0.014
		Exponential	0.01, 10.04, 0.017	0.35, 2.25, 0.105	0.00, −1.06, 0.010	0.00, −24.56, 0.011	0.13, −0.63, 0.005
OS	Short	Linear	0.76, −10.92, 0.127^†^	0.14, −7.22, 0.623	0.00, −14.27, 0.975	0.00, −36.43, 0.940	0.44, −14.99, 0.337
		Quadratic	0.77, −9.00, 0.483	0.99, −32.47, 0.031^†^	0.24, −13.34, 0.874^†^	0.92, −44.39, 0.287^†^	0.93, −21.09, 0.272^†^
		Sigmoid	0.76, −7.86, 0.033	0.00, −5.25, <0.001	0.00, −11.43, 0.005	0.00, −32.68, <0.001	0.46, −12.27, 0.002
		Gaussian	0.76, −7.91, 0.033	0.15, −4.40, 0.097	0.00, −11.51, 0.005	0.00, −33.62, 0.062	0.45, −12.20, 0.058
		DoG	0.73, −7.74, <0.001	0.31, −4.95, <0.001	0.00, −11.55, 0.903	0.00, −32.41, 0.99	0.48, −12.27, 0.324
		Exponential	0.77, −10.19, <0.001	0.15, −6.40, 0.006	0.00, −13.60, 0.005	0.00, −35.34, 0.002	0.46, −14.21, 0.002
	Long	Linear	0.96, −10.03, 0.021	0.85, −12.03, 0.077	0.63, −13.44, 0.208	0.21, −33.91, 0.547	0.08, −5.67, 0.713
		Quadratic	0.99, −15.13, 0.084^†^	0.91, −12.13, 0.296^†^	0.64, −11.53, 0.604^†^	0.47, −33.52, 0.729^†^	0.55, −6.56, 0.668^†^
		Sigmoid	0.98, −8.46, 0.028	0.89, −10.50, 0.109	0.63, −10.02, 0.061	0.31, −30.98, 0.047	0.23, −2.90, 0.108
		Gaussian	0.97, −5.06, 0.029	0.88, −10.18, 0.110	0.64, −10.09, 0.060	0.48, −4.95, 0.041	0.57, −5.21, 0.081
		DoG	0.86, −2.37, <0.001	0.78, −7.73, <0.001	0.65, −10.95, 0.192	0.20, −30.53, 0.554	0.02, −2.42, 0.123
		Exponential	0.98, −11.57, <0.001	0.89, −12.27, 0.007	0.62, −12.50, 0.002	0.20, −33.31, 0.002	0.08, −5.12, 0.009

^†^Best curve fit for the respective plot.

The results for the metrics over AD/short SRTs suggest that there was not enough time to inhibit the distractor and resolve the target–distractor competition (Fig. 7*A*), consistent with saccade trajectories shifted toward the distractor with positive deviations. The initial angle and end point deviation, reflecting the overall saccade vector, exhibited weighted averaging of the two object locations where distance decreased the weight of the distractor. Curvature measures reflected a different process wherein the strength of the distractor in producing a curved trajectory followed an increase-to-plateau pattern, indicative of an increasing effect of the distractor on saccade curvature that maxes out by 67.5° (AD = 3). This result shows that although the maximum possible curvature increases as AD increases (when the objects are farther away from each other, there is more space for the saccade to curve toward the distractor), the maximum observed curvature stays the same from AD 3 to 5. For long SRTs, target–distractor competition was completed, resulting in saccade trajectories shifted away from the distractor with negative deviations. Varying AD resulted in DoG-shaped curves for all metrics, which is indicative of a spatial suppressive surround where there was the most suppression of the distractor at 67.5° (AD = 3; Fig. 7*B*).

### Similarity effects

**Figure 8.**
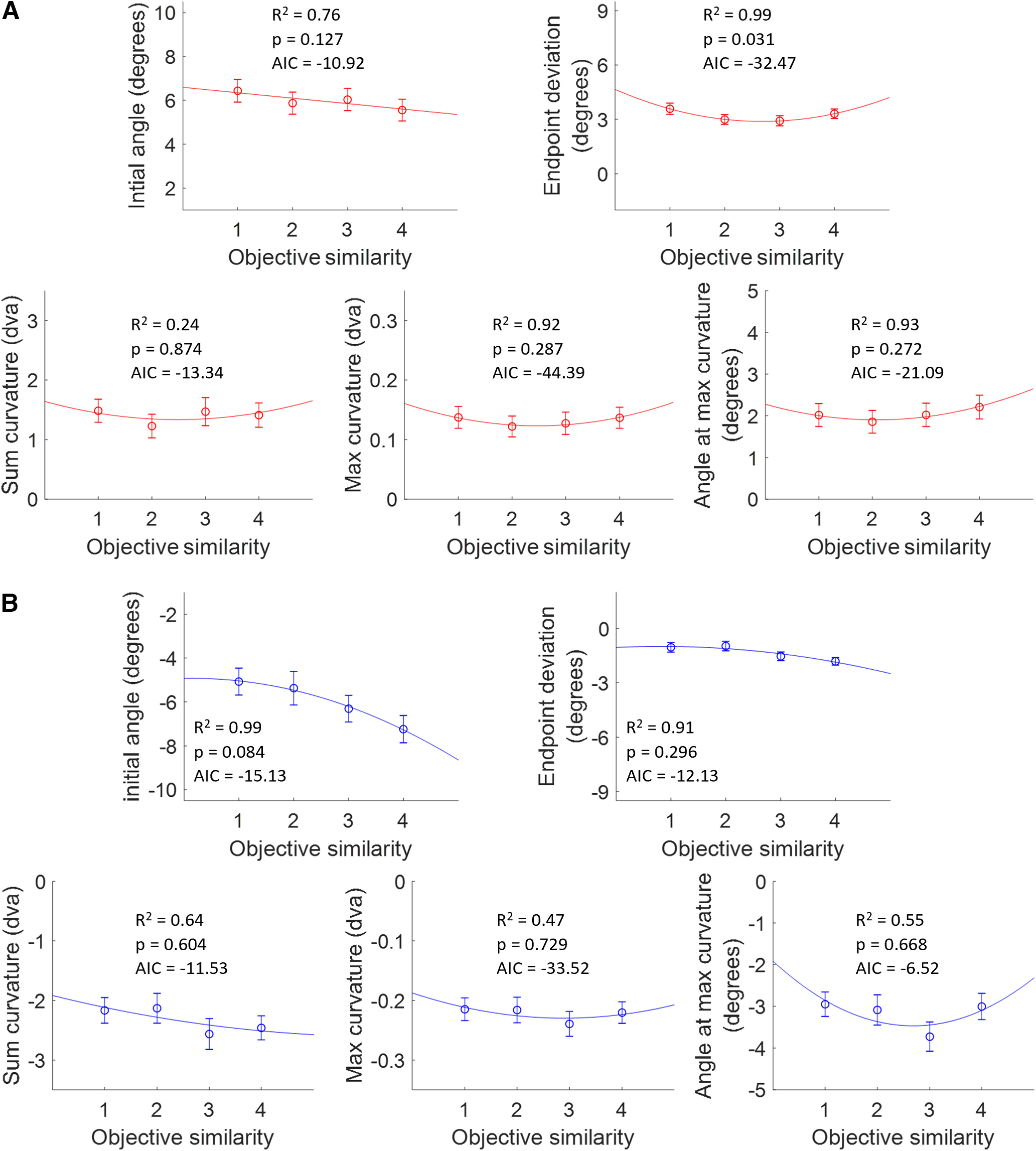
Saccade metric averages over OS with curve fits for short and long SRTs. Metric values were averaged across all angular distances. Curve fits were chosen according to goodness-of-fit metrics (*R*^2^, AIC, *p*). Plots include the respective *R*^2^, *p*-values, and AIC. Error bars are SEM. ***A***, Metrics over OS for short SRTs. Curve fits were all quadratic except linear for the initial angle. ***B***, Metrics over OS for long SRTs. Curve fits were all quadratic.

We repeated these analyses focusing on the effects of similarity. The average metric values were analyzed against OS for short and long SRTs, collapsing over AD (Fig. 8). For short SRTs, there were no main effects of OS on the metrics. For long SRTs, there was a main effect of OS on end point deviation (Table 2), but there were no significant differences across OS levels according to Scheffé’s test *post hoc* analysis. There were no significant interaction effects between OS and AD for short or long SRTs.

We again fit a range of functions to the metrics plotted against OS. The best curve fits according to goodness-of-fit measures (Table 4) for short and long SRTs were used (Fig. 8*A*,*B*). For short SRTs (Fig. 8*A*, Table 4), only the quadratic fit for end point deviation had a significant *p*-value (*F* test from the regression analysis).

For OS/short SRTs, end point deviations were significantly affected by similarity (Fig. 8*A*). In general, at short SRTs there was not enough time to fully resolve the target–distractor competition and inhibit the distractor, although the significant quadratic fit (*p* = 0.031) found for end point deviation suggests that an object-based suppressive surround modulates the average saccade vector during the active discrimination process. For OS/long SRTs, we did not see any significant effects of similarity on saccade trajectories (Fig. 8*B*).

### Similarity–angular distance split plots

**Figure 9.**
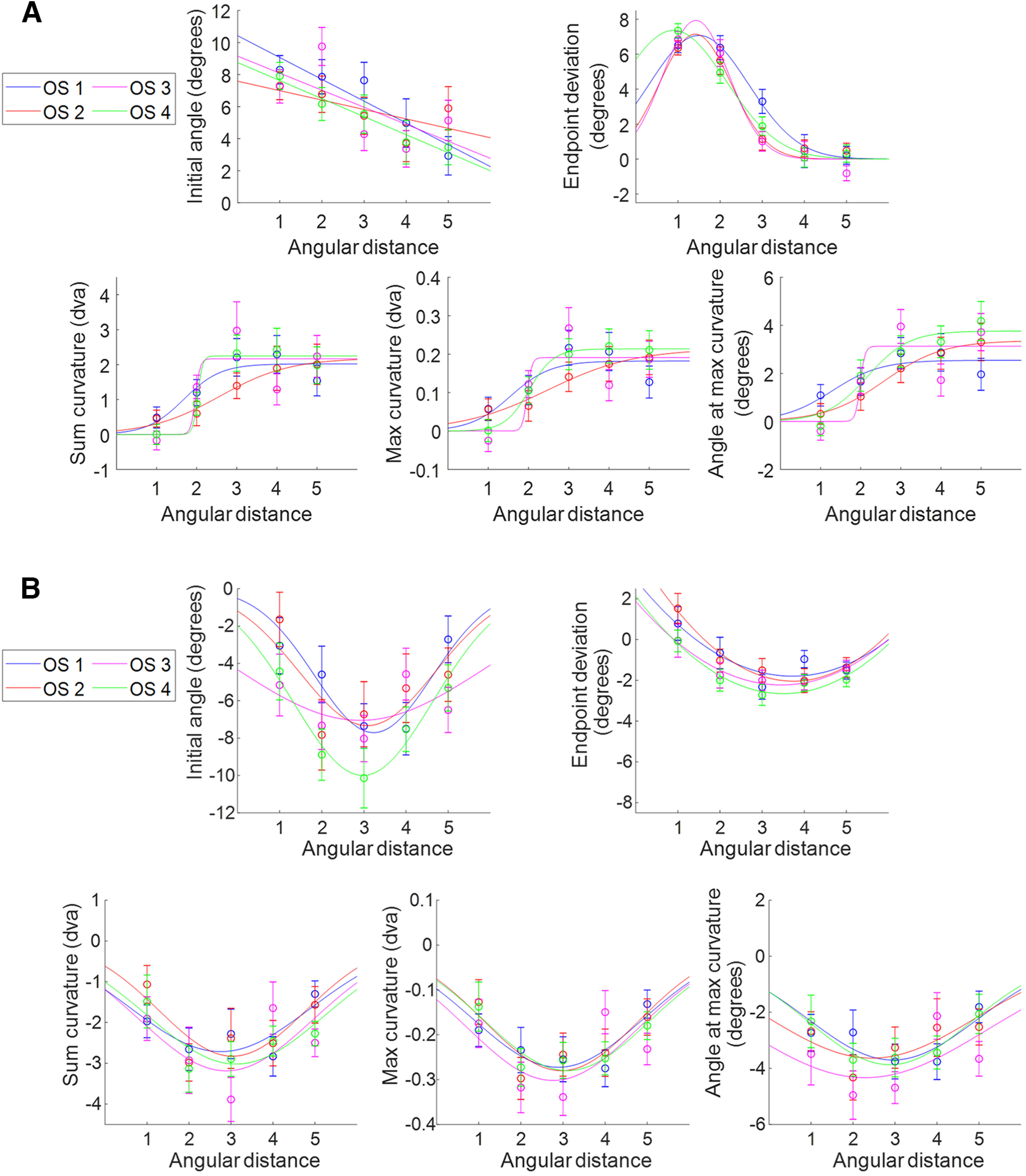
Metrics over AD with separate lines for OSs 1–4, plotted for short and long SRTs. Each plot was fit with the respective best fit per metric from the average metric plots. ***A***, Metrics over AD with split OS lines for short SRTs. ***B***, Metrics over AD with split OS lines for long SRTs.

To better investigate the relationship between OS level and AD, we plotted the average metric values across AD with separate lines for each OS level (Fig. 9). The overall patterns match those of the averaged metric plots from Figure 7, *A* and *B*. We fit each line with the best curve fit from the prior AD analysis for their respective SRT periods. For both short and long SRTs, the best fit of the average metric plots (Table 5) matched well to the individual similarity lines, with most curves showing significant fits (*p* < 0.05) or trends (*p* < 0.1).

**Table 5:** *R*^2^, AIC, *p*-values for each curve fitted to metric plots over AD separated by OS for short and long SRTs

SRT period	Metric (curve fit)	OS 1	OS 2	OS 3	OS 4
Short	Initial angle (Linear)	0.87, −1.05, 0.021*	0.45, 3.20, 0.217	0.43, 9.49, 0.223	0.96, −6.93, 0.004
	End point deviation (Gaussian)	0.99, −10.86, 0.002*	0.98, −3.56, 0.012*	0.98, −1.34, 0.015*	0.99, −10.41, 0.003*
	Sum curvature (Sigmoid)	0.81, −4.75, 0.045*	0.98, −16.18, 0.008*	0.74, 0.39, 0.024*	0.98, −12.38, <0.001*
	Max curvature (Sigmoid)	0.66, −26.16, 0.077*	0.96, −38.48, 0.009*	0.75, −20.40, 0.025*	0.99, −45.25, 0.001*
	Angle at max curvature (Sigmoid)	0.67, −2.21, 0.045*	0.99, −21.03, 0.001*	0.75, 7.54, 0.027*	0.93, −2.18, 0.028*
Long	Initial angle (Gaussian)	0.88, 18.55, 0.026*	0.62, 9.47, 0.078*	0.20, 8.21, 0.048*	0.94, 0.57, 0.007*
	End point deviation (Quadratic)	0.76, −0.92, 0.240	0.97, −9.15, 0.031*	0.96, −13.45, 0.037*	0.92, −7.74, 0.080*
	Sum curvature (Gaussian)	0.63, −4.22, 0.031*	0.70, −2.89, 0.043*	0.32, 2.54, 0.087*	0.67, −4.06, 0.026*
	Max curvature (Gaussian)	0.77, −30.34, 0.018*	0.73, −27.56, 0.031*	0.34, −21.21, 0.085*	0.85, −32.20, 0.012*
	Angle at max curvature (Gaussian)	0.64, −1.10, 0.031*	0.51, −0.35, 0.035*	0.23, 5.50, 0.074*	0.94, −10.63, 0.004*

*Significant effects (*p* < 0.05) or significant trends (*p* < 0.1).

**Figure 10.**
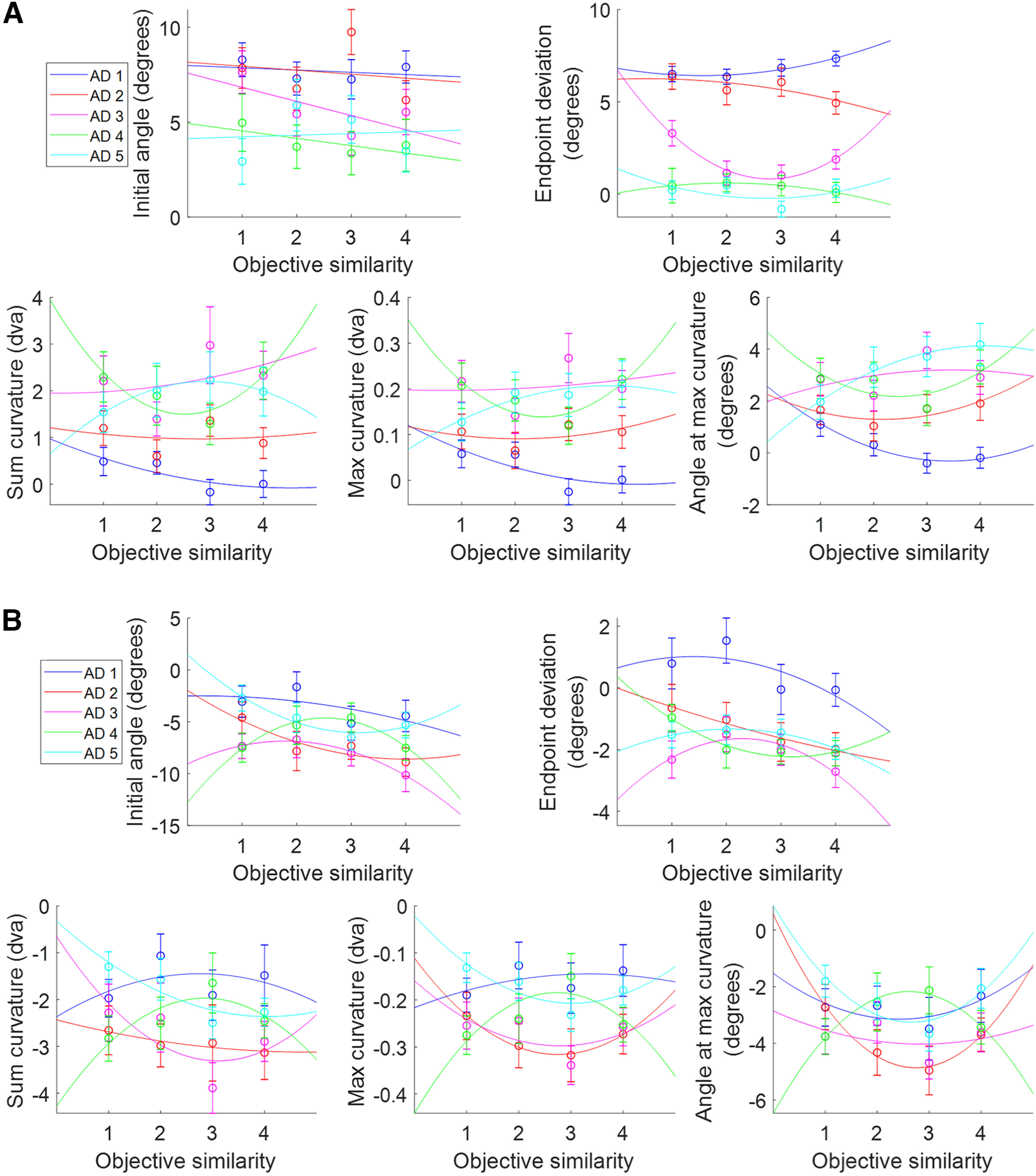
Metrics over OS with separate lines for ADs 1–5, plotted for short and long SRTs. Each plot was fit with the respective best fit per metric from the average metric plots. ***A***, Metrics over OS with split AD lines for short SRTs. ***B***, Metrics over OS with split AD lines for long SRTs.

The same was done for the average metric values across OS with separate lines for AD (Fig. 10). For both short and long SRTs, the best fit of the average metric plots (Table 6) did not match well to the individual AD lines, with only four curves showing significant trends (*p* < 0.1). This further suggests that OS is not as strong as AD in affecting saccade metrics, as was previously reflected in Figures 6, 7, and 8, and that AD and OS do not interact in the target–distractor competition that produces deviations in saccade trajectories.

**Table 6:** *R*^2^, AIC, *p*-values for each curve fitted to metric plots over OS separated by AD for short and long SRTs

SRT period	Metric (curve fit)	AD 1	AD 2	AD 3	AD 4	AD 5
Short	Initial angle (linear)	0.09, −3.09, 0.692	0.03, 6.36, 0.828	0.47, 2.95, 0.311	0.52, −2.93, 0.289	0.01, 5.45, 0.918
	End point deviation (quadratic)	0.97, −15.33, 0.184	0.67, −3.44, 0.572	0.98, −11.38, 0.125	0.99, −30.03, 0.057*	0.228, −0.46, 0.878
	Sum curvature (quadratic)	0.70, −8.87, 0.549	0.02, −3.87, 0.992	0.154, 0.711, 0.920	0.76, −6.22, 0.491	0.99, −22.93, 0.107
	Max curvature (quadratic)	0.66, −24.93, 0.587	0.016, −25.64, 0.992	0.038, −18.95, 0.981	0.73, −25.23, 0.516	0.88, −30.06, 0.353
	Angle at max curvature (quadratic)	0.97, −12.75, 0.168	0.64, −7.06, 0.600	0.15, 1.60, 0.922	0.48, −0.87, 0.720	0.98, −11.66, 0.133
Long	Initial angle (Gaussian)	0.42, 6.18, 0.761	0.83, 2.53, 0.409	0.99, −10.65, 0.097*	0.96, −4.90, 0.196	0.94, −2.54, 0.251
	End point deviation (quadratic)	0.57, −0.66, 0.656	0.97, −13.00, 0.173	0.92, −10.77, 0.279	0.95, −11.73, 0.226	0.99, −24.68, 0.093*
	Sum curvature (Gaussian)	0.15, −2.70, 0.924	0.83, −15.13, 0.414	0.53, −0.67, 0.683	0.68, −5.18, 0.566	0.83, −6.72, 0.415
	Max curvature (Gaussian)	0.28, −24.55, 0.847	0.99, −42.03, 0.079*	0.31, −21.67, 0.830	0.66, −22.65, 0.584	0.74, −25.89, 0.508
	Angle at max curvature (Gaussian)	0.44, −3.16, 0.751	0.99, −12.28, 0.123	0.12, 0.37, 0.938	0.98, −12.28, 0.155	0.75, −2.34, 0.497

*Significant trends (*p* < 0.1).

### Initial angle and end point deviation versus vector average

**Figure 11.**
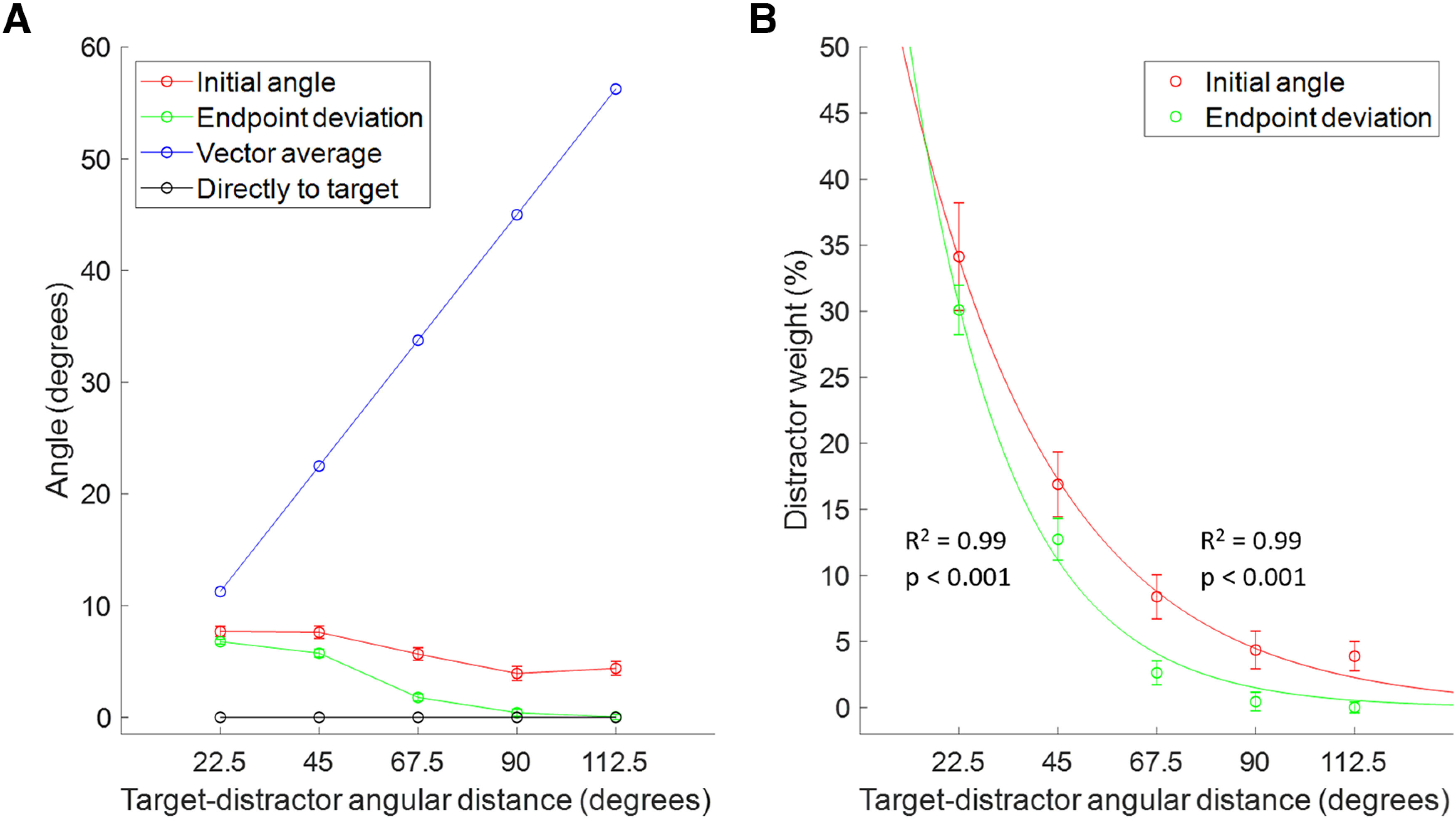
Initial angle and end point deviation at short SRTs versus vector average and direct to target (winner-take-all). ***A***, Average initial angle and end point deviation compared with the vector average between the target and distractor, and the direct-to-target trajectory over angular distance in degrees. A saccade made directly to the distractor would fall on the *y*-axis. ***B***, Distractor weight based on initial angle and end point deviation values over angular distance in degrees. Both sets of data were fitted with an exponential curve.

As mentioned above, Figure 7*A* shows that initial angle and end point deviation for short SRTs reflected the effects of weighted vector averaging, where the distractor lost weight as AD increased. Here, we compared the process of dynamically weighted vector averaging to vector averaging assuming equal weights. In Figure 11*A*, we plotted the average values for initial angle and end point deviation from Figure 7*A* and compared them with the predicted vector average of the target and distractor locations as AD increases, placing a 50:50 weight on each object. For comparison, we plotted the angles for a trajectory made directly to the target (lone target with no distractor) that would occur in a winner-take-all process. The initial angle and end point deviation, while similar to each other over AD, do not follow the same trend as the predicted vector average. This supports the idea that they are based instead on a weighted vector average of the target and distractor. In Figure 11*B*, we show the weighting of the distractor by dividing the initial angle and end point deviation by the predicted vector average, respectively, and multiplying by 50% for even weighting. We found that these percentages were well fit with an exponential curve (*R*^2^ = 0.99; *p* < 0.001 for both fits), showing that the weight of the distractor exponentially decreases as it gets further away from the target (as AD increases).

## Discussion

We examined the effects of varying the AD and OS of complex target and distractor objects on saccade trajectories made to the target during a delayed match-to-sample task to elucidate how perceptual and spatial relationships are encoded by the oculomotor system. The time between display onset and saccade initiation influenced saccade direction, prompting us to further analyze the effects of angular distance and similarity based on SRT. While some prior studies have shown differential effects of distractor attraction and repulsion based on broad timing differences (Theeuwes and Godijn, 2004; McSorley et al., 2006; Walker et al., 2006; Mulckhuyse et al., 2009; Hickey and van Zoest, 2012), we determined the time course of the switch from distractor attraction to repulsion for each of the five saccade trajectory metrics. The effects of SRT on the saccade metrics reflect the development of the competition process between saccade goals; more time between target–distractor onset and saccade initiation resulted in more time to resolve competition and inhibit the distractor. When there was not enough time to resolve the target–distractor competition, there was an effect of angular distance on saccade trajectories, consistent with saccade vector averaging and incomplete winner-take-all (Port and Wurtz, 2003). With increasing SRT, distance modulated the saccade metrics via putative mechanisms producing a spatial suppressive surround. Varying target–distractor similarity had limited effects, only influencing end point deviations at short SRTs that reflected an object-based suppressive surround during the active discrimination phase of competition.

### Effects of saccadic reaction time on saccade direction

Although we did not purposefully manipulate the SRT, the time taken to decide which object was the target and to initiate a saccade naturally varied across trials because of participants taking more or less time to discriminate complex objects based on the difficulty of each trial. This is dependent on the timing of the target–distractor competition process, the time it takes to select a target and inhibit the distractor, ∼200–220 ms (McSorley et al., 2006). With a shorter SRT period (∼100–220 ms), the target–distractor competition remained unresolved at saccade initiation, resulting in curvature toward the distractor that reflects weighted vector averaging between the target and distractor. With a longer SRT period (∼260–500 ms), there was enough time to inhibit the distractor, causing saccades to curve away from the distractor. In the time between the short and long SRTs (∼220–260 ms), the distractor was in a transition period between competing with the target and being inhibited.

However, the transition period ranges varied between each saccade metric. The curvature metrics (sum curvature, max curvature, and angle at max curvature) reached the midpoint of the transition period earlier than the angle metrics (initial angle and end point deviation), suggesting that after competition resolves and the process flips to distractor inhibition, saccade curvature is affected sooner than the initial angle and end point deviation. This is likely because the curvature metrics reflect the residual effects of an incomplete winner-take-all process. Initial angle and end point deviation occurred later because they are reflective of weighted vector averaging in saccade planning, based on that prior winner-take-all process. These findings for saccade angle and curvature are consistent with prior studies that found the initial speed and direction of smooth pursuit of a moving target against a moving distractor reflected weighted vector averaging, until the winner-take-all process completed (>300 ms; Ferrera and Lisberger, 1995, 1997; Lisberger and Ferrera, 1997; Recanzone and Wurtz, 1999). In a saccade task with two spots at varying spatial separations, longer saccadic reaction times occurred when the distances were larger (Ottes et al., 1984). Similar to pursuit programming, saccade vector averaging occurred for trials with the shorter SRTs, whereas longer SRTs (>300 ms) were associated with a winner-take-all process (Ottes et al., 1984). These smooth pursuit and saccade studies showed a winner-take-all process needing ∼300 ms, which is consistent with our results as the transition zones completed in <300 ms for every metric.

### Effects of angular distance on saccade trajectories

For short SRTs, initial angle and end point deviation decreased with increasing angular distance, reflective of weighted vector averaging of saccade plans before movement initiation. The saccade angle was shifted more toward the target with increasing distance, suggesting that when planning a saccade to one location, competing plans have decreased strength the further away they are to the saccade goal. As the same pattern was seen for both initial angle and end point deviation, they must both be derived from the underlying saccade vector. Our results further support a prior proposal that the initial direction and end point deviation of a saccade are correlated (Van der Stigchel et al., 2007), refuting other models that propose end point deviation is independent of the online correction (curvature) guiding the saccade back toward the target (McSorley et al., 2004).

The curvature metrics showed evidence of an incomplete winter-take-all process developed from saccade goal competition that had not had enough time to resolve. As angular distance increased, saccade curvature increased and then plateaued. Although the maximum possible saccade curvature is limited by the angular distance between target and distractor for deviations toward the distractor (there is no such limit for deviations away from the distractor), we found that the maximum curvature is reached at 67.5° (AD = 3). This maximum is maintained up through a target–distractor distance of 112.5° (AD = 5), the largest distance in our study. Beyond that range, it is likely to decrease again [e.g., when approaching 180° of separation (opposite vectors)]. The effects on saccade curvature reflect incomplete winner-take-all competition between saccade goals, whereas initial angle and end point deviation reflected a weighted vector average for saccade motor plans to target and distractor locations.

For long SRTs, initial angle, end point deviation, and the curvature metrics shifted away from the distractor in a DoG manner as distance increased, consistent with a spatial suppressive surround in oculomotor planning as was predicted and found in visual processing by the selective tuning model of attention (Cutzu and Tsotsos, 2003; Hopf et al., 2006; Kehoe et al., 2018a; Yoo et al., 2022). When the distractor was close to the target (AD = 1, 2), it was in the attentional window of the target and less suppression was induced. At the medium distance from target (AD = 3), the distractor exhibited peak suppression from the suppressive surround gradient. Finally, as the distractor moved outside of the suppressive zone surrounding the attended target (AD = 4, 5), suppression was weaker and curvature similarly lessened. While spatial suppressive surrounds have been found in early and intermediate visual areas (Müller and Kleinschmidt, 2004; Hopf et al., 2006), our results extend this to saccade trajectories derived from the spatial locations of complex objects. Therefore, oculomotor planning is dependent on an attentional priority map with weighted representations of target and distractor locations.

### Effects of similarity on saccade trajectories

We split our similarity results by short and long SRTs to assess how similarity affects the competition phase separately from the distractor inhibition phase. For short SRTs, there were no statistically significant differences between similarity levels when examining the angle and curvature metrics over increasing OS (as the objects became less similar). However, there was a significant DoG-shaped pattern for end point deviation, consistent with a nonspatial suppressive surround found for simple features such as color, orientation, and direction of motion (Tsotsos et al., 2005; Tombu and Tsotsos, 2008; Störmer and Alvarez, 2014; Yoo et al., 2018; Kehoe et al., 2018a). Our results suggest, for the first time, that an object-based suppressive surround may be active during the discrimination phase of target–distractor competition. For long SRTs, there were no effects of similarity on saccade metrics, suggesting that object representations only mattered during the discrimination phase of target–distractor competition. Further research is needed to better understand object space suppressive surrounds and to determine whether object-based similarity can modulate saccade metrics.

While we hypothesized that the effects of similarity on saccade trajectories would be modulated by distance through a multiplicative gain mechanism (Connor et al., 1997; Salinas and Abbott, 1997; McAdams and Maunsell, 1999; Treue and Martínez-Trujillo, 1999; Salinas and Thier, 2000), we found no such interaction. This suggests that spatial layout and object similarity are independently processed and incorporated into oculomotor planning. The stage of the target–distractor competition process gives rise to independent sequential suppressive surround effects first through similarity then distance.

### Oculomotor circuitry and target selection

This study demonstrates that varying the distance and similarity of complex objects, as well as the observed variability in timing of the competition process, affect target selection and saccade planning differently. Saccades initiated during the early stages of target–distractor competition (short SRTs) exhibit weighted vector averaging caused by enhanced neural activity of oculomotor neuron populations encoding the competing saccade goals (Findlay and Walker, 1999; Godijn and Theeuwes, 2002; Port and Wurtz, 2003). The final motor plan is a result of reweighting potential saccade goals based on their behavioral relevance and priority, which for this task is the similarity of the objects in question to the previewed target (Kehoe et al., 2018b). Online corrections (evidenced by saccade curvatures) pull the overall saccade trajectory back toward the target since its initial vector is more toward the distractor because of the weighted average of the object locations. With more time (long SRTs), the target–distractor competition resolves, consistent with the population of neurons encoding the unchosen saccade goal decreasing in activity (McPeek et al., 2003; McPeek, 2006; White et al., 2012), producing curvatures away from the distractor. A spatial suppressive surround develops around the attended target, further modulating the effect of the distractor on the saccade vector.

The spatial layout of objects in the visual field is encoded in the oculomotor system through attentional priority maps where neurons encoding objects of interest (through both bottom-up and top-down features) have increased activity (Fecteau and Munoz, 2006; Fernandes et al., 2014; White et al., 2017). With sufficient time, the suppressive zone surrounding the attended target develops in the oculomotor system. Studies have shown a link between spatial attention and oculomotor areas (Moore and Fallah, 2001, 2004; Bisley and Goldberg, 2003; Moore et al., 2003; Krauzlis et al., 2013), which suggests that spatial surround suppression that developed in visual processing areas feeds into attentional priority maps in the oculomotor system.

In contrast, the target–distractor similarity in this delayed match-to-sample task only affected saccade trajectories while the discrimination process was active. Complex visual information is processed in later regions of the ventral stream (Gross, 1992), such as areas TE and TEO (Baylis and Rolls, 1987; Miller et al., 1993; Tanaka, 2003). While our target and distractor are processed and discriminated in these higher-order visual regions, our results show that their output projects to the oculomotor system for saccade planning and distractor inhibition. Once the discrimination process completes, similarity no longer affects saccade planning. Therefore, spatial layout and object similarity are processed in their respective visual processing areas, with distance and similarity information independently feeding into different aspects of oculomotor planning.

### Applications to decision-making models

Decision-making models define the functions of the active decision-making process and exit points at which the decision is made. These two stages, active and complete, were clearly distinguishable from their effects on saccade trajectories. Our results show that there are two distinct stages in which saccades can be made. When the target–distractor discrimination process is active, as evidenced by short SRTs, saccades curve toward the distractor. When it is complete, with long SRTs, saccades curve away from the distractor. The range of SRTs and resultant effects on saccade trajectories in our task suggest that the decision-making process can be completed after enough evidence has been accumulated (complete discrimination) or exited early before a complete decision has been made (incomplete discrimination). These clear effects on saccade trajectories can be applied to decision-making models, such as the recognition-primed decision model (Klein, 1999), recognition/metacognition model (Cohen et al., 1996), and OODA (observe-orient-decide-act) loop (Boyd, 2018), where quick decisions are made because of time constraints. The overall shape (amount and direction of saccade curvature) acts as a measure of how far along in the decision-making process the participant was at the point of saccade initiation (the exit point). Switching from manual to saccade responses in decision-making tasks can result in the ability to determine the stage at which the decision-making process was exited (early or after a complete decision is reached) and distinguish between these phases on a trial-by-trial basis.

### Conclusion

We varied distance and similarity in a saccadic response, visual search task using novel complex objects. We found that the saccade metrics distinguished between active and complete decision-making processes, where distractor inhibition affected saccade curvatures sooner than saccade vector angles, suggesting that these are independent processes with separate priority maps. Distance had a strong influence on saccade trajectories in both stages, but the effect of similarity was limited to the active discrimination decision-making process. During this active stage, the effects of distance on saccade metrics followed spatial averaging and incomplete winner-take-all. When discrimination completed and the distractor was inhibited, a spatial suppressive surround mediated the effects of distance on saccade vector angles and curvatures. In comparison, we found evidence suggesting that an object space suppressive surround mediated target–distractor similarity during the active discrimination process. We did not find any interaction between spatial and complex object discriminations, suggesting that distance and similarity are processed separately, consistent with spatial processing in the dorsal stream and object processing in the ventral stream. Distance and similarity differences must then independently feed into the oculomotor system for final saccade plans to be created. These results suggest that saccade responses would be more beneficial than manual responses in decision-making studies to allow for determining at what point in the decision-making process a decision was made based on these saccade trajectory metrics.

## Synthesis

Reviewing Editor: Nicholas J. Priebe, University of Texas at Austin Decisions are customarily a result of the Reviewing Editor and the peer reviewers coming together and discussing their recommendations until a consensus is reached. When revisions are invited, a fact-based synthesis statement explaining their decision and outlining what is needed to prepare a revision will be listed below. The following reviewer(s) agreed to reveal their identity: NONE.

Synopsis

Two reviewers and the reviewing editor evaluated the manuscript “Target-distractor competition modulates saccade trajectories in space and object-space” and all reviewers agreed that the manuscript provides a solid examination of how saccade metrics reveal the sensorimotor decision process. The authors demonstrate that saccades to targets bend depending on the presence of distractors and reveal some of the features that influence this bend, including the latency of the saccade, the distance between the target and distractor, and, to a less extent, the similarity of the target and distractor. These results are useful for modeling the decision processes that underlie accurate eye movements in cluttered environments. There was consensus, both in the reviews and in the consultations that followed, that additional analyses are needed and some clarifications of the experimental details are necessary. The largest issue that the reviewers raised, however, is the interpretation of the effects of object similarity on saccades. The reviewers felt that the present manuscript does not demonstrate a clear, statistically significant, effect of target/distractor similarity on saccades. These issues and others are detailed below, which you should find useful in revising your manuscript, if you decide to resubmit it to eNeuro or another journal.

Major Issues:

1) Object similarity: A major concern with this manuscript is that there is little to no effect observed for the object similarity (OS) experiments. The best case, best fit results in a trend with a p = 0.084. That could be by chance, since so many metrics and fits were examined, or maybe there is just not enough data. Based on a qualitative view of the data, there is potentially a trend of a greater suppressive effect with greater distractor differences. If we are to believe this effect is real though, it only happens with the initial saccade angle and potentially only at particular distances. The latter possibility would mean that OS depends on distance meaning they are not really independent parameters-i.e., you only observe a dependence on object similarity at distances (ADs) of 2 and 3 degrees (Fig. 7B). It would be really helpful if there were a figure similar to Figure 7 looking at the OS curves at different ADs. It was unclear if Figs. 5 and 6 were averaged across all OSs and ADs, respectively. An overall concern is that the experimental design for object similarity is not adequately capturing an object-dependence effect. It might be that even an OS = 1 is already an obvious difference for the human visual system.

2) Presentation: The presentation of results could be more engaging. The results should be explained in more detail, since many times, the description jumps ahead without really explaining how we ended up getting there or what we are really looking at. At times, it reads like a recitation of statistics. It’s a good idea to remind the reader of the question or hypothesis that addressed by each analysis. It may help to include a basic figure showing actual individual saccade traces and all the measurements and fits and why they were chosen. For example, it is not always clear what 0 means in the figures. Does negative mean that the saccade is moving away from the target and distractor, while positive values mean it is moving away from the target, but towards the distractor. If this is true, then the abstract should say away and towards the distractor, since in both cases, saccades are then deviating away from the target, initially. Summary statistics and test results can be reported in tables. If the numbers are reported in a table, they don’t need to be reproduced in the text.

3) Time course of attraction/repulsion: One of the key results in this study is that the effect of a distractor shifts from attraction to repulsion. The authors show this by separating the data into two reaction time ranges, short and long. It would also be useful to plot the time course of this effect. Included in such a time course should be a distribution, so that it is clear whether there is repulsion, attraction or the lack of an effect.

4) Terminology: “Repulsion” is a more accurate term than “inhibition” to describe the distractor effect for longer RTs. “Attraction” and “repulsion” are what happens to the saccade trajectory. Inhibition of the distractor is the putative mechanism. Thus “inhibition” is not complementary to “attraction.” It gets a bit confusing because there is reference to “suppressive surrounds,” but it seems like these might be better described as repulsive surrounds. In any case, the authors should be clear in their terminology when referring to empirical observations vs imputed underlying mechanisms.

5) Gradient plots: The gradient plots may be redundant with other representations of the data. Furthermore, they may be misleading in that there is no evidence that the quantities represented are circularly symmetric. If either is the case, please remove.

Minor Issues:

1) The distance effects are clear, but it might be worthwhile to also plot the average initial angle versus the predicted vector average for each angular distance to strengthen that conclusion.

2) Line 22. “Thus, saccade responses are more beneficial than manual responses in studies of decision-making models.” Not sure this comparison is helpful. Each paradigm has its strengths.

3) Line 133. What was the duration of the preview?

4) Line 236 “saccadic frequency” might be better described as a “saccade latency distribution” putting the emphasis more on the explanatory variable. Also, it would be interesting to see the distribution of latencies for single target (no distractor) trials as the difference between simple and choice reaction times might be an index of decision time.

5) Line 225. “saccade target onset asynchrony” should probably be defined in the Methods. “Split saccade reaction times” is not a good phrase. A reader might think this refers to the reaction time of something called a “split saccade.” It would be better to use a phrase like ’division of saccade reaction times” or “classification of saccade reaction times.”

6) Line 245. Does this refer to Fig 4.

7) Line 293 It should be noted that the maximum possible curvature increases with target-distractor distance. How was this taken into account?

8) The work of Ottes et al showing a time-dependent shift from averaging to winner-take-all saccades is highly relevant and should be discussed/cited. https://doi.org/10.1016/0042-6989(84)90172-X

## Author Response

Response:

We thank the reviewers and the reviewing editor for the feedback and the opportunity to improve the manuscript accordingly.

Major Issues:

1) Object similarity: A major concern with this manuscript is that there is little to no effect observed for the object similarity (OS) experiments. The best case, best fit results in a trend with a p = 0.084. That could be by chance, since so many metrics and fits were examined, or maybe there is just not enough data. Based on a qualitative view of the data, there is potentially a trend of a greater suppressive effect with greater distractor differences. If we are to believe this effect is real though, it only happens with the initial saccade angle and potentially only at particular distances. The latter possibility would mean that OS depends on distance meaning they are not really independent parameters-i.e., you only observe a dependence on object similarity at distances (ADs) of 2 and 3 degrees (Fig. 7B). It would be really helpful if there were a figure similar to Figure 7 looking at the OS curves at different ADs. It was unclear if Figs. 5 and 6 were averaged across all OSs and ADs, respectively. An overall concern is that the experimental design for object similarity is not adequately capturing an object-dependence effect. It might be that even an OS = 1 is already an obvious difference for the human visual system.

Response:

Thank you for the suggestions. We removed all discussion of the trend in initial angle at long SRTs (p = 0.084) throughout the Results and Discussion. That focus on long SRTs/initial angles may have detracted from the effect of objective similarity at short SRTs when the discrimination of target and distractor was active: we found a significant curve fit (p = 0.031) for endpoint deviation over OS at short SRTs (Figure 8A). This is the result we initially referenced when 2 suggesting the existence of an object-based suppressive surround during the active phase of target-distractor competition. We softened the language throughout the manuscript regarding the existence of an object-based suppressive surround from the result on this single metric to ensure it is depicted as a possible conclusion from our findings and that more research would need to be done to further explore this possibility (Lines 578, 681).

We added in a figure looking at the OS curves at different ADs (Figure 10). As well, we added the respective table of goodness of fit metrics (Table 6). When going back to our analysis for Figure 9, we noticed that the data used for the short SRTs plot was based on a range of short SRTs from an earlier analysis rather than the current metric-specific ranges defined in the manuscript. We updated this plot (Figure 9) and Table 5 using the correct SRT ranges.

Correcting the analysis to use the accurate ranges of SRTs did not change the results for this analysis (all values that were significant in Table 5 in the initial submission are still significant with this update). We checked all other analyses to ensure the correct ranges were used, and this section was the only error. We apologize for the mistake.

We edited the figure legends for Figures 7 and 8 to clarify how they were averaged across all OSs and ADs, respectively, as they were averaged across all OSs (for Figure 7) and ADs (for Figure 8).

Addressing: “An overall concern is that the experimental design for object similarity is not adequately capturing an object-dependence effect. It might be that even an OS = 1 is already an obvious difference for the human visual system.”

Work on low level features, such as color or orientation showed that similarity affected oculomotor planning for even very obvious differences such as the congruent color effect (Ludwig and Gilchrist, 2003; Mulckhuyse et al., 2009). In a study investigating the timecourse of distractor inhibition (Kehoe et al., 2018a), similar complex object stimuli were used and effects of similarity on oculomotor planning were found. We based our choice of stimuli building upon this paper. We addressed this concern and clarified this point in the Introduction (Line 58).

Due to additions to the Introduction from the revisions described in this response, some additional small changes were made to the Introduction to ensure it stayed within the word limit of 750 words.

To summarize, there was evidence for object-based suppressive surrounds for endpoint deviation over objective similarity at short SRTs. This is consistent with a suppressive surround in object-space inhibiting the distractor during the active phase of target-distractor competition (before competition completes) when it is slightly different from the target in appearance, causing saccade trajectories to land closer to the target at these similarity levels. However, as it was only found in one metric, rather than a pattern across metrics, we have drawn speculations from it rather than definitive conclusions. Additional research will be needed to investigate this further. 3

2) Presentation: The presentation of results could be more engaging. The results should be explained in more detail, since many times, the description jumps ahead without really explaining how we ended up getting there or what we are really looking at. At times, it reads like a recitation of statistics. It’s a good idea to remind the reader of the question or hypothesis that addressed by each analysis. It may help to include a basic figure showing actual individual saccade traces and all the measurements and fits and why they were chosen. For example, it is not always clear what 0 means in the figures. Does negative mean that the saccade is moving away from the target and distractor, while positive values mean it is moving away from the target, but towards the distractor. If this is true, then the abstract should say away and towards the distractor, since in both cases, saccades are then deviating away from the target, initially.

Summary statistics and test results can be reported in tables. If the numbers are reported in a table, they don’t need to be reproduced in the text.

Response:

We added in sentences to the beginning and end of each results section to remind the reader of the hypotheses addressed in each section as suggested. As well, we removed most statistics and test results from the text and reported them in a table (Table 3). In addition, we added p-values to Table 4 to ensure the goodness of fit metrics mentioned here matched what is provide on the plots in Figures 7 and 8. One minor typo was noticed in the initial angle over AD, Long SRTs p-value within the plot in Figure 7. This was fixed and we apologize for the error.

We included a figure to show the measurements of each saccade metric on an example saccade trajectory (Figure 3B) and a separate figure of individual saccade traces coloured by SRT (Figure 3A). We discussed these analyses and clarified why the metrics were used in the Results section “Individual saccade traces and saccade metric measurements”.

We clarified what 0 means in the Methods (Line 175) and clarified that positive and negative mean toward the distractor and away from the distractor, respectively, throughout the manuscript. We added arrows in Figure 4 pointing up/down to show that positive = towards and negative = away, and we added “Towards Distractor” and “Away from Distractor” into the x-axis labels of Figures 3 and 6 to ensure these directions are clear and to show that the towards/away terminology is in relation to the distractor and not the target. The towards/away terminology was also added to the Abstract in Lines 11 and 15.

3) Time course of attraction/repulsion: One of the key results in this study is that the effect of a distractor shifts from attraction to repulsion. The authors show this by separating the data into two reaction time ranges, short and long. It would also be useful to plot the time course of this effect. Included in such a time course should be a distribution, so that it is clear whether there is repulsion, attraction or the lack of an effect.

Response: 4

We added a plot of the time course of the metrics (Figure 4) with t-test results comparing the metric values over STOA to zero (representative of the distribution of effect at a given time bin). This now clearly depicts our method of separating the data into short and long reaction time ranges (and a middle transition range where metric values were not significantly different than zero) given in Table 1. We used up/down arrows to clarify the distinction between attraction and repulsion as being positive and negative values.

4) Terminology: “Repulsion” is a more accurate term than “inhibition” to describe the distractor effect for longer RTs. “Attraction” and “repulsion” are what happens to the saccade trajectory. Inhibition of the distractor is the putative mechanism. Thus “inhibition” is not complementary to “attraction.” It gets a bit confusing because there is reference to “suppressive surrounds,” but it seems like these might be better described as repulsive surrounds. In any case, the authors should be clear in their terminology when referring to empirical observations vs imputed underlying mechanisms.

Response:

We adjusted our terminology to “repulsion” instead of “inhibition” throughout the manuscript, as well as “attraction”, when describing what happens to the saccade trajectory. As well, we described the suppressive surrounds as “putative mechanisms producing suppressive surrounds”.

We continued to use the term “suppressive surround” rather than “repulsive surround” in the manuscript as it is the terminology used in the relevant prior research including both behavioral (Cutzu and Tsotsos, 2003; Hopf et al., 2006) and neuroimaging (Boehler et al., 2009) studies. We included the suggested terminology in the Introduction (Line 47 and 66) and Discussion (Line 476) to clarify these points.

5) Gradient plots: The gradient plots may be redundant with other representations of the data.

Furthermore, they may be misleading in that there is no evidence that the quantities represented are circularly symmetric. If either is the case, please remove.

Response:

We removed these figures since there is no evidence that the quantities represented are circularly symmetric.

Minor Issues:

1) The distance effects are clear, but it might be worthwhile to also plot the average initial angle versus the predicted vector average for each angular distance to strengthen that conclusion.

Response:

We plotted the average initial angle and endpoint deviation over AD from short SRTs versus the predicted vector average (and direct to target trajectory angles) for each angular distance to show 5 that those metrics are reflective of weighted vector averaging where the weight on the distractor decreases with increasing distance (Figure 11A). Figure 11B shows the weighting based on our data for initial angle and endpoint deviation at short SRTs as percentages, and we fitted the data with exponential decay functions to show this decrease in weight on the distractor as distance increases. We made a new section in the results to describe these plots and explain the findings (Results section “Initial angle and endpoint deviation versus vector average”).

As well, we wanted to note that we changed a couple instances in the manuscript of “endpoint deviation angle” to “endpoint deviation” to remain consistent with our choice of wording throughout.

2) Line 22. “Thus, saccade responses are more beneficial than manual responses in studies of decision-making models.” Not sure this comparison is helpful. Each paradigm has its strengths.

Response:

We removed this line from the Abstract.

3) Line 133. What was the duration of the preview?

Response:

The preview was under participant control, so we clarified this in the methods stating “The target object was then previewed on the screen (Figure 2A) until the participant pressed the button to indicate they were ready to begin the trial. Upon button press, the preview was replaced with a central fixation cross”.

4) Line 236 “saccadic frequency” might be better described as a “saccade latency distribution” putting the emphasis more on the explanatory variable. Also, it would be interesting to see the distribution of latencies for single target (no distractor) trials as the difference between simple and choice reaction times might be an index of decision time.

Response:

We changed all instances of “saccade frequency” to “saccade latency distribution” and adjusted the plot axis label to reflect this. We also added the saccade latency distribution for single target trials to this plot and found that it peaks at ∼150ms with a steeper decline over time than for trials with a distractor.

5) Line 225. “saccade target onset asynchrony” should probably be defined in the Methods. “Split saccade reaction times” is not a good phrase. A reader might think this refers to the reaction time of something called a “split saccade.” It would be better to use a phrase like “division of saccade reaction times” or “classification of saccade reaction times.”

Response: 6

We added a new section in the Methods called “Saccade target onset asynchrony” to define this term. As well, we changed “split saccade reaction times” to say “classification of” as suggested.

In addition, the results section “Saccade target onset asynchrony and classification of saccadic reaction times” was rearranged and reworded in some areas to accommodate the new and revised figures and to clarify the analyses and plots.

6) Line 245. Does this refer to Fig 4.

Response:

This line referred to what is now Figure 4B in the revised manuscript. We changed this line to say, “We used these results to classify trials by their SRTs into short, long, and transition periods (Figure 4B) where significant positive deviations towards the distractor occurred for short SRTs, and significant negative deviations away from the distractor occurred for long SRTs.”

As well, we made a new plot with significant deviations towards and away from the distractor (t-test versus zero) shown to further demonstrate the metric-specific classifications of saccade reaction times into short, long, and transition periods (Figure 4A).

7) Line 293 It should be noted that the maximum possible curvature increases with target-distractor distance. How was this taken into account?

Response:

We added this consideration into the manuscript in the Results (Lines 311, 315, and 351) and

Discussion (Line 532). The maximum possible curvature was not taken into account when designing the experiment or running analyses, although our results suggest a more complex integration of distance (maximum occurs at 67.5 degrees for short SRTs, spatial suppressive surround for long SRTs). While the maximum possible curvature increases with increasing AD, we were looking to see if it was vector averaged (linear with target-distractor distance), maximum asymptote at some specific distance, or if there was no effect at a large enough distance. The curvatures did not reach, or come close to, the limit of the maximum possible curvature defined by target-distractor distance. It is also important to note that the maximum possible curvature is only a one-sided limit, as it would only affect curvature towards the distractor (at short SRTs). There is no such limit for curvature away from the distractor (at long SRTs).

8) The work of Ottes et al showing a time-dependent shift from averaging to winner-take-all saccades is highly relevant and should be discussed/cited. https://doi.org/10.1016/0042- 6989(84)90172-X

Response:

We added this reference into the Discussion and briefly discussed the study (Line 507).
